# QTL meta-analysis in *Arabidopsis* reveals an interaction between leaf senescence and resource allocation to seeds

**DOI:** 10.1093/jxb/eru125

**Published:** 2014-04-01

**Authors:** Fabien Chardon, Sophie Jasinski, Monique Durandet, Alain Lécureuil, Fabienne Soulay, Magali Bedu, Philippe Guerche, Céline Masclaux-Daubresse

**Affiliations:** ^1^UMR1318 INRA AgroParisTech, Institut Jean-Pierre Bourgin, INRA Versailles, Route Saint Cyr, 78026 Versailles Cedex, France

**Keywords:** Leaf senescence, nitrogen and carbon allocation, harvest index, yield, flowering time.

## Abstract

Sequential and monocarpic senescence are observed at vegetative and reproductive stages, respectively. Both facilitate nitrogen (N) remobilization and control the duration of carbon (C) fixation. Genetic and environmental factors control N and C resource allocation to seeds. Studies of natural variation in *Arabidopsis thaliana* revealed differences between accessions for leaf senescence phenotypes, seed N and C contents, and N remobilization efficiency-related traits. Here, a quantitative genetics approach was used to gain a better understanding of seed filling regulation in relation to leaf senescence and resource allocation. For that purpose, three *Arabidopsis* recombinant inbred line populations (*Ct-1*×*Col-0*, *Cvi-0*×*Col-0*, *Bur-0*×*Col-0*) were used to map QTL (quantitative trait loci) for ten traits related to senescence, resource allocation, and seed filling. The use of common markers across the three different maps allowed direct comparisons of the positions of the detected QTL in a single consensus map. QTL meta-analysis was then used to identify interesting regions (metaQTL) where QTL for several traits co-localized. MetaQTL were compared with positions of candidate genes known to be involved in senescence processes and flowering time. Finally, investigation of the correlation between yield and seed N concentration in the three populations suggests that leaf senescence disrupts the negative correlation generally observed between these two traits.

## Summary

Mapping of metaQTL controlling leaf senescence and seed resource allocation in *Arabidopsis* reveals that leaf senescence might disrupt the general negative correlation observed between yield and seed nitrogen concentration.

## Introduction

As the last stage of leaf development, senescence is mainly characterized by the loss of green pigments, chloroplast degradation, and reduced protein content in leaves. Nutrient transport from senescing leaves to the surviving structures is known as nutrient remobilization. Because both a decrease in photosynthesis and nutrient remobilization occurs in leaves at this developmental stage, leaf senescence has a strong impact on important agronomical traits such as seed yield, as well as seed protein and lipid contents. For this reason, leaf senescence has been widely studied in plants, especially crops, for a long time (Thomas and Stoddart, 1980). Studies showing an association between senescence and crop productivity are numerous and were reviewed recently by Gregersen *et al.* (2013). Selection and breeding of plants showing delayed senescence in the field, especially when water is limited during the post-anthesis period, have been largely undertaken with the aim to increase grain yield (Borrell *et al.*, 2000). However, the negative relationship between grain protein content and yield that was reported in various cereals suggests that delaying leaf senescence to increase grain yield has a negative effect on grain protein content (Simmonds, 1995; Oury and Godin, 2007; Blanco *et al.*, 2012). To date, the relationship between leaf senescence, and yield or grain protein content has been studied mainly in cereals, such as maize, wheat, rice, or barley. There are almost no reports on dicots, with the exception of a few studies on rapeseed, cowpea, and soybean (Abu-Shakra *et al.*, 1978; Ismail *et al.*, 2000; Hunkovà *et al.*, 2011).

Leaf senescence is not a passive decay. Its initiation, the duration of its execution phase, and the onset of its terminal phase, which ends with death, are regulated through tight genetic control. Factors influencing the different senescent phases are numerous and they are both endogenous and exogenous. It is known, for example, that leaf senescence is accelerated under mild stress conditions such as a lack of fertilization or an excess of shading. Plant hormones also play a role, delaying senescence in the case of cytokinins (Gan and Amasino, 1997; Zavaleta-Mancera *et al.*, 1999) or inducing premature senescence as triggered by ethylene treatments (Grbic and Bleecker, 1996; Schippers *et al.*, 2007).

The transcription factors involved in controlling leaf senescence have been extensively investigated and several members of the NAC (NAM, NO APICAL MERISTEM; ATAF1/2, ARABIDOPSIS TRANSCRIPTION ACTIVATION FACTOR 1/2; CUC2, CUP-SHAPED COTYLEDON 2) and WRKY families have been identified for their roles in leaf senescence (Fischer, 2012 for a review). WRKY53 is the best understood senescence-related WRKY, and interaction of the *WRKY53* promoter with the MEKK1 kinase suggests that the WRKY53 signalling pathway cascade integrates both senescence and stress responses (Miao *et al.*, 2004; Miao *et al.*, 2008; Zentgraf *et al.*, 2010). Among the long list of NAC transcription factors involved in plant senescence (Olsen *et al.*, 2005), the wheat (*Triticum turgidum* ssp. *durum*) NAC gene termed *TtNAM-B1* was found through an extensive QTL (quantitative trait loci) study of cereal grain protein content (Uauy *et al.*, 2006). The transcript level of *TtNAM-B1* was shown to increase in flag leaves during senescence, and gene silencing of *TtNAM-B1* in wheat led to plants with both a stay-green phenotype and lower grain protein content. One of the *Arabidopsis* homologues of *TtNAM-B1, AtNAP* (*ANAC029*), is also induced during leaf senescence (Guo and Gan, 2006). However the role of *AtNAP* in grain filling and yield has not yet been described in *Arabidopsis*. Besides AtNAP, other *NAC* genes such as *AtNAC2* (or *ANAC092* also known as *ORE1, ORESARA 1* which means long-living in Korean) have been reported to play a role in the control of leaf senescence (Kim *et al.*, 2009; Balazadeh *et al.*, 2010). Studies of *ORE1* provided the first evidence of epigenetic control of leaf senescence by microRNA. Indeed, *MiR164b* influences *ORE1* transcript levels (Kim *et al.*, 2009). Other epigenetic and posttranscriptional mechanisms regulating leaf senescence (Tian and Chen, 2001; Ay *et al.*, 2009) show the complexity of the regulatory pathways involved.

With the large number of factors and signals influencing leaf senescence, we have decided to investigate leaf senescence traits using quantitative genetics approaches (Diaz *et al.*, 2006; Wingler *et al.*, 2010). In our laboratory, studies on the natural variation of leaf senescence and nitrogen remobilization traits led us to consider the links between nitrogen use efficiency traits, yield, plant biomass, and leaf senescence events before and after flowering in *Arabidopsis* (Diaz *et al.*, 2005; Diaz *et al.*, 2006; Masclaux-Daubresse and Chardon, 2011). Diaz *et al.* (2006) showed that leaf senescence observed at the early vegetative developmental stage was positively correlated with amino acid content in the rosette and negatively with rosette dry weight. This result was the first evidence of a negative link between plant biomass and leaf senescence before flowering in *Arabidopsis*. Further studies using a set of five recombinant inbred lines (RILs) of the *Bay-0*×*Shahdara* population confirmed that differential leaf senescence was associated with differential biomass and amino acid content (Diaz *et al.*, 2005). Using ^15^N-tracing experiments, Diaz *et al.* (2008) demonstrated that the severity of leaf senescence symptoms is positively correlated with ^15^N remobilization efficiency at the vegetative stage. In contrast, a later study of 19 *Arabidopsis* accessions at the reproductive stage (Masclaux-Daubresse and Chardon, 2011) showed that there is no link between leaf senescence scores and the efficiency of nitrogen remobilization to seeds even though nitrogen-remobilization efficiency correlated strongly with the harvest index.

Previous studies on natural variation of *Arabidopsis thaliana* have also revealed differences between accessions for leaf senescence phenotypes (Balazadeh *et al.*, 2008) as well as for N remobilization efficiency-related traits and yield (Masclaux-Daubresse and Chardon, 2011). However the information obtained on RILs or accessions were fragmentary and sometimes contradictory.

The aim of the present study was to investigate the links between leaf senescence, yield, and seed filling in *Arabidopsis* to determine whether, as seems to be the case in crops, traits are linked and controlled by some common genetic basis. We used three *Arabidopsis* recombinant inbred line populations optimized for QTL mapping, with *Col-0* as a common parent (Simon *et al.*, 2008), to map QTL for traits related to senescence, resource allocation, and seed yield. These populations were chosen as the parental lines display contrasting leaf senescence phenotypes (Balazadeh *et al.*, 2008), and/or N remobilization efficiencies (Masclaux-Daubresse and Chardon, 2011). The use of common markers across the three different maps facilitated QTL meta-analysis approaches (Chardon *et al.*, 2004; Sosnowski *et al.*, 2012) leading to the identification of the most interesting regions where QTL co-localize. MetaQTL and candidate gene positions are discussed. Finally, the relationship between seed N concentrations and yield regarding leaf senescence is analysed in *Arabidopsis*.

## Materials and methods

### Plant material and growth conditions

A subset of 154 RILs from the *Ct-1*×*Col-0* population, 164 RILs from the *Cvi-0*×*Col-0* population, and 164 RILs from the *Bur-0*×*Col-0* population (Simon *et al.*, 2008), in addition to the four parental lines (*Ct-1*, *Cvi-0*, *Bur-0*, and *Col-0*) were used in this study. The three accessions crossed to *Col-0* were rationally chosen from a core collection that was previously defined to maximize the genetic and phenotypic diversity in a reduced number of accessions (McKhann *et al*. 2004). Seeds were obtained from the Versailles Biological Resource Centre for *Arabidopsis* (http://publiclines.versailles.inra.fr/). Seeds were sown on damp Whatman filters, stratified for three d at 4 °C and then transferred to a growth cabinet under long-day conditions at 21 °C for 2 d. Three seedlings (with emerging radicle) per genotype were planted in soil in 7-cm pots and transferred to a non-heated and naturally lit greenhouse to be vernalized from November 2010 to February 2011 (mean temperature was 5.5 °C). After eight weeks, one plantlet per pot was randomly retained without phenotype selection. After 12 weeks of vernalization, plants were transferred to a growth chamber under long-day conditions (16/8h photoperiod at 150 mmol photons m^–2^ s^–1^); 21 °C day temperature and 18 °C night temperature; relative humidity of 65%. From this time, three times a week the plant trays were moved around the growth chamber to reduce position effects. The plants were no longer watered once the oldest siliques had turned yellow. At this stage, bags were put over the plants to prevent seed dispersion. Plants were kept in the growth chamber until dry and then harvested. Three replicates were grown for each RIL and ten for the parental lines.

### Seed composition analyses (Seed C% and Seed N%)

Near-infrared spectroscopy was used to determine the seed composition. Spectra were acquired on approximately 100mg of intact seeds with a Fourier transform near-infrared (FTNIR) analyser (Antaris II spectrometer; Thermofisher Scientific, France). Seed carbon and nitrogen concentrations were estimated using developed NIRS calibration models.

### Statistical analyses

Statistical analyses of traits were carried out on the mean of three replicates using XLSTAT (http://www.xlstat.com). Correlation coefficients were calculated using Pearson’s correlations. The coefficient of variation (CV) was computed as the ratio of the standard deviation to the mean.

### QTL detection

Composite interval mapping was carried out using the R/QTL library in the R environment (Arends *et al.*, 2010). A backward regression method was used for the genome scan. To identify an accurate significance threshold for each trait, an empirical threshold was determined using 1000 permutations (Churchill and Doerge, 1994). QTL positions were assigned to relevant regions at the point of the maximum likelihood odds ratio (LOD). QTL confidence intervals (CI) were calculated by 1.5 LOD drop from the maximum LOD position.

### Map projection and QTL meta-analyses

Meta-analysis was performed to combine QTL information from independent experiments and refine chromosomal intervals when all collected QTL were projected onto the consensus map. For the same chromosome across multiple populations, a consensus map was constructed from the three population maps using BioMercator version 3.0 software (Sosnowski *et al.*, 2012) as described by Arcade *et al.* (2004). All QTL identified for the ten traits in individual populations using R/QTL were projected onto the consensus map separately. Information on the original chromosomal position, confidence interval (CI), and proportion of phenotypic variance (*R* ^*2*^) explained by each QTL (as summarized in Supplementary Table S1 available at *JXB* online) were used for the projection. For each chromosome, meta-analysis was used to estimate the number, positions, and 95% confidence interval of the metaQTL using BioMercator version 3.0 software (Sosnowski *et al.*, 2012). The meta-analysis first determines the best model based on the following criteria: AIC (Akaike information criterion), AICc, AIC3, BIC (Bayesian information criterion), and AWE (average weight of evidence). The best QTL model was selected when values of the model selection criteria were the lowest in at least three of the five models. Consensus QTL from the optimum model are regarded as metaQTL. The effect of metaQTL in each original population was estimated by the phenotypic difference between the two genotypes at the marker closest to the metaQTL.

## Results

### Analysis of ten traits related to leaf senescence and resource allocation in three *Arabidopsis* RIL populations and the corresponding parental accessions

**Fig. 1.**
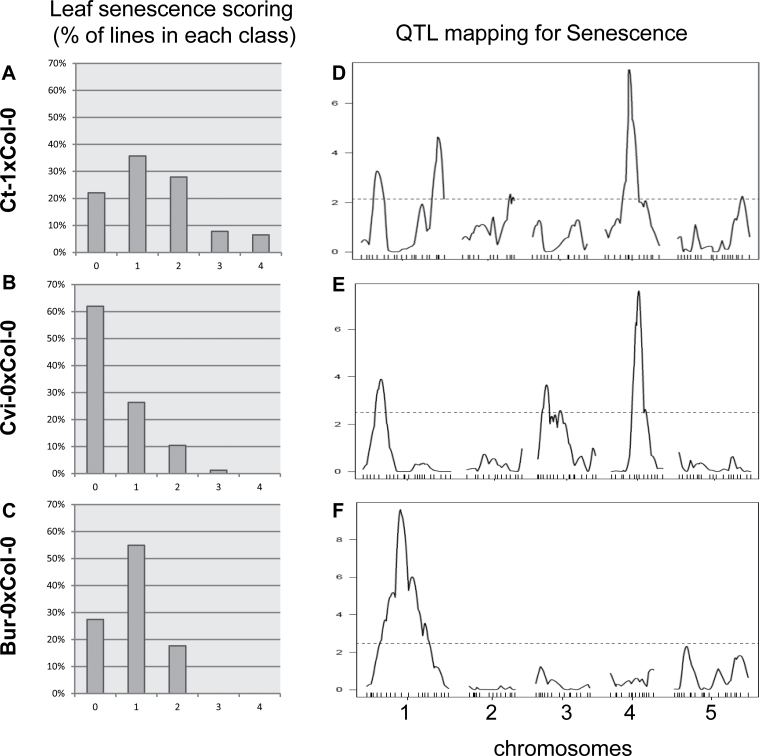
Phenotypic variation and corresponding QTL mapping for leaf senescence in the three RIL populations, *Ct-1*×*Col-0* (A, D), *Cvi-0*×*Col-0* (B, E), and *Bur-0*×*Col-0* (C, F). (A–C) Distribution of RILs among the five classes of senescence, from score 0 for fully green plants to score 4 for yellow rosettes, in the three RIL populations. (D–F) QTL mapping for leaf senescence. LOD score curves for the five chromosomes are shown. Each peak above the threshold (dashed line corresponding to 2.3 LOD) identifies a QTL.

Ten traits were measured or computed from measured traits to characterize leaf senescence and resource allocation (Table 1) in three RIL populations (*Ct-1*×*Col-0*, *Cvi-0*×*Col-0*, *Bur-0*×*Col-0*) and the corresponding parental accessions. The harvest index (HI, the ratio between seed dry weight and total plant dry weight [Seeds + Stem + Rosette]) is commonly used to estimate grain productivity per plant. Here, we defined the RV ratio (the ratio between reproductive organ weight [Stem + Seeds] and vegetative organ weight [Rosette]) as an indicator of the partition of biomass produced before and after flowering. The parental lines were highly contrasted for most of the studied traits, and *Col-0* showed an intermediate phenotype within the range of the three others accessions (Table 2). The *Ct-1* accession was characterized by earlier leaf senescence compared with the other three accessions. At harvest, the biomass before flowering of *Cvi-0* and *Ct-1* was lower than that of *Col-0* and *Bur-0*, with earlier flowering time (FT) as well as a higher HI and RV. *Cvi-0* seeds had low carbon content (Seed C%) and high nitrogen content (Seed N%) compared with the other three accessions. All ten traits showed continuous variation in the three RIL populations. Transgressive segregation in both directions was recorded for most of the traits, indicating the presence of favourable alleles in both parents (see Mean, Max, and Min values of RILs in Table 2). The three studied populations also showed different ranges of variation for the ten traits. For example, variation in leaf senescence scores was widespread in the *Ct-1*×*Col-0* population, whereas in the *Bur-0*×*Col-0* population it was low, and in the *Cvi-0*×*Col-0* population no yellow leaves were observed for most of lines (Table 2; Fig. 1 A–C). The CVs were all very high, with 68–143% for leaf senescence, highlighting the huge phenotypic variation in the populations. CVs were higher for leaf senescence, Rosette, Stem, Seeds, RV, FT, and Seed N% in the *Cvi-0*×*Col-0* population, but higher for HI, TGW, and Seed C% in the *Bur-0*×*Col-0* population.

**Table 1.** Measured or computed traits

Trait name	Kind	Phenotyping scoring
Leaf senescence	Measured	Scores of leaf senescence before flowering time by visual phenotyping of leaf yellowing, from score 0: fully green plants to score 4: yellow rosette (Diaz *et al.* 2006)
Flowering time	Measured	Number of d following stratification to opening of first flower
Rosette	Measured	Dry rosette weight at harvest (mg/plant)
Stem	Measured	Inflorescence dry weight measured as the weight of stem and silique envelopes at harvest (mg/plant)
Seeds	Measured	Seed yield measured as the weight of all dry seeds (mg/plant)
HI	Computed	Ratio between Seeds and total plant dry weight (Seeds + Stem + Rosette)
RV	Computed	Ratio between reproductive organ weight (Stem + Seeds) and vegetative organ weight (Rosette)
Seed C%	Measured	Carbon percentage (g. (100g dry matter)^–1^) in seeds estimated by NIRS
Seed N%	Measured	Nitrogen percentage (g. (100g dry matter)^–1^) in seeds estimated by NIRS
TGW	Measured	Thousand grain (seed) weight (mg/plant)

**Table 2.** Descriptive statistics of traits in the three RIL populations and the parental lines For each RIL population, mean and SD of each trait is calculated from all the RILs including three plant repeats per RIL.

Traits^1^	RIL populations																		Parental lines			
	*Ct-1*×*Col-0*						*Cvi-0*×*Col-0*						*Bur-0*×*Col-0*									
	Mean	Min	Max	SD	CV		Mean	Min	Max	SD	CV		Mean	Min	Max	SD	CV		*Col-0*	*Ct-1*	*Cvi-0*	*Bur-0*
Leaf senescence	1.4	0	4	1.11	0.79		0.5	0	3	0.73	1.43		1.0	0	2	0.66	0.68		0.3	1.6	0.0	0.6
Rosette	265	65	533	87	0.33		205	31	608	131	0.64		411	57	799	120	0.29		377	188	41	405
Stem	2128	908	3199	479	0.23		1617	631	2809	465	0.29		2347	877	3576	432	0.18		2302	1883	576	2546
Seeds	1454	614	2241	339	0.23		1056	326	1888	334	0.32		1243	78	1874	367	0.30		1583	1570	465	1412
HI	0.38	0.22	0.43	0.03	0.09		0.37	0.24	0.43	0.04	0.10		0.30	0.05	0.43	0.07	0.23		0.38	0.44	0.45	0.34
RV	14.8	7.3	31.2	4.19	0.28		17.8	5.9	48.6	8.67	0.49		9.5	4.8	31.0	3.05	0.32		10.4	18.8	27.1	9.9
FT	21.0	16	35.7	3.01	0.14		15.4	5	40.0	7.15	0.46		28.3	21	42.0	2.93	0.10		23.9	16	7	27.4
TGW	19	16	25	2	0.08		23	16	30	3	0.14		24	15	37	4	0.15		17	20	27	27
Seed C%	58.0	56.2	59.4	0.58	0.01		56.5	53.8	58.5	0.84	0.01		57.2	54.7	59.4	0.83	0.01		57.2	58.8	56.3	57.3
Seed N%	4.00	3.58	4.65	0.17	0.04		4.59	3.82	5.48	0.30	0.06		4.26	3.69	4.78	0.21	0.05		4.15	3.89	4.87	4.45

^1^Traits are described in Table 1. HI, harvest index; RV, ratio between reproductive and vegetative organ weights; FT, flowering time; TGW, thousand grain weight. Min: minimal value of the trait in the population; Max: maximal value of the trait in the population; SD: standard deviation; CV: coefficient of variation. For parental lines, only means are shown (*n*=10).

### Genetic correlation between leaf senescence and resource allocation traits was observed in the three populations

In the three populations, Rosette, Stem, and Seeds were strongly and positively correlated with each other (Table 3), showing that the variation in plant biomass between RILs is uniformly distributed among the three organs. The HI was highly positively correlated to Seeds showing that HI variation is mainly due to grain yield variation. The RV was negatively correlated to both Rosette and FT, suggesting that RV is a good indicator of the partition of biomass produced before and after flowering. The trait Seeds was positively correlated with Seed C% in both the *Cvi-0*×*Col-0* and *Bur-0*×*Col-0* populations confirming that, as described for many crops, carbon fixation is a major limiting factor for yield. Surprisingly, this correlation was negative for the *Ct-1*×*Col-0* population. Seed C% and Seed N% were negatively correlated in all populations suggesting that seed filling with either proteins or lipids is antagonistic. As a result, Seeds was negatively correlated with Seed N% in *Cvi-0*×*Col-0* and *Bur-0*×*Col-0*, but not in *Ct-1*×*Col-0*. Despite these differences, the HI was positively correlated with Seed C% and negatively correlated with Seed N% in the three populations.

**Table 3.** *Pearson correlation coefficients between traits in each RIL population (light grey for* Ct-1×Col-0, *mid grey for* Cvi-0×Col-0 *and dark grey for* Bur-0×Col-0*)* For each correlation pair, only significant correlation coefficients are shown (*P*<0.05). *ns* for non-significant

Trait^1^	Leaf Senescence	Stem	Seeds	Rosette	HI	RV	FT	Seed C%	Seed N%	TGW
Leaf Senescence		–0.65	–0.60	–0.54	*ns*	0.21	*ns*	0.39	–0.35	*ns*
		*ns*	*ns*	*ns*	*ns*	*ns*	*ns*	*ns*	*ns*	*–0.23*
			*ns*	–0.25	*ns*	0.26	–0.42	*ns*	*ns*	*–0.36*
Stem	–0.65		0.80	0.64	–0.28	*ns*	*ns*	–0.49	0.39	*0.32*
	*ns*		0.86	0.78	–0.21	–0.62	0.62	*ns*	–0.28	*–0.33*
	*ns*		0.31	0.36	*ns*	*ns*	–0.28	*ns*	*ns*	*ns*
Seeds	–0.60	0.80		0.59	0.33	*ns*	*ns*	–0.24	*ns*	*ns*
	*ns*	0.86		0.71	0.28	–0.56	0.52	0.31	–0.43	*–0.26*
	*ns*	0.31		0.53	0.85	–0.25	–0.47	0.62	–0.54	*ns*
Rosette	–0.54	0.64	0.59		ns	–0.75	0.27	–0.55	0.40	*ns*
	ns	0.78	0.71		–0.26	–0.81	0.76	*ns*	*ns*	*–0.48*
	–0.25	0.36	0.53		0.30	–0.76	*ns*	0.37	*ns*	*0.38*
HI	*ns*	–0.28	0.33			*ns*	*ns*	0.48	–0.41	*–0.22*
	*ns*	–0.21	0.28	–0.26		0.23	–0.29	0.40	–0.32	*0.24*
	*ns*	*ns*	0.85	0.30		–0.27	–0.36	0.62	–0.53	*ns*
RV	0.21	*ns*	*ns*	–0.75	*ns*		–0.38	0.35	–0.24	*ns*
	*ns*	–0.62	–0.56	–0.81	0.23		–0.75	0.23	*ns*	*0.47*
	0.26	*ns*	–0.25	–0.76	–0.27		–0.31	–0.25	*ns*	*–0.31*
FT	*ns*	*ns*	*ns*	0.27	*ns*	–0.38		*ns*	*ns*	*ns*
	*ns*	0.62	0.52	0.76	–0.29	–0.75		–0.22	*ns*	*–0.52*
	–0.42	–0.28	–0.47	*ns*	–0.36	–0.31		–0.24	0.28	*0.47*
Seed C%	0.39	–0.49	–0.24	–0.55	0.48	0.35	*ns*		–0.86	*ns*
	*ns*	*ns*	0.31	*ns*	0.40	0.23	–0.22		–0.88	*0.29*
	*ns*	*ns*	0.62	0.37	0.62	–0.25	–0.24		–0.85	*0.30*
Seed N%	–0.35	0.39	*ns*	0.40	–0.41	–0.24	*ns*	–0.86		*ns*
	*ns*	–0.28	–0.43	*ns*	–0.32	*ns*	*ns*	–0.88		*ns*
	*ns*	*ns*	–0.54	*ns*	–0.53	*ns*	0.28	–0.85		*ns*
TGW	*ns*	0.32	*ns*	*ns*	–0.22	*ns*	*ns*	*ns*	*ns*	
	–0.23	–0.33	–0.26	–0.48	0.24	0.47	–0.52	0.29	*ns*	
	–0.36	*ns*	*ns*	0.38	*ns*	–0.31	0.47	0.30	*ns*	

^1^Traits are described in Table 1. HI, harvest index; RV, ratio between reproductive and vegetative organ weights; FT, flowering time; TGW, thousand grain weight.

With regards to the leaf senescence trait, different correlation patterns were also found for the three populations. In the *Ct-1*×*Col-0* population, leaf senescence was negatively correlated with plant biomass (Rosette, Stem, and Seeds) and Seed N%, and positively correlated to RV and Seed C% (Table 3). In the *Bur-0*×*Col-0* population, leaf senescence was also negatively correlated to FT and positively to the RV. In the *Cvi-0*×*Col-0* and *Bur-0*×*Col-0* populations, leaf senescence was negatively correlated to TGW.

### QTL analyses in the three RIL populations

**Fig. 2.**
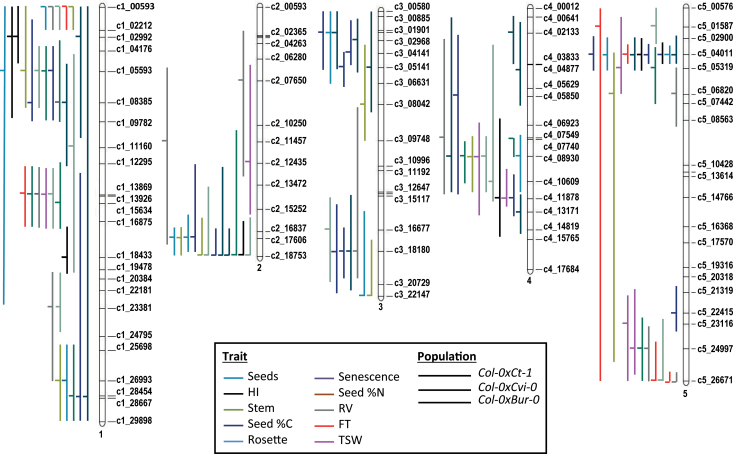
Mapping of 101 QTL detected for leaf senescence and for traits related to resource allocation, onto a single consensus map. Each QTL is shown by a horizontal line, indicating the most likely position, with a vertical line indicating the confidence interval around this position. Line style indicates the population where the QTL was detected. Line colour shows the trait affected. Numbers on the right correspond to the genetic markers used to establish the consensus genetic map (Simon *et al.*, 2008).

The genetic architecture of the studied traits was addressed with a QTL approach using the R/QTL library in the R environment (Arends *et al.*, 2010). Variation between RILs allowed us to map QTL for the ten studied traits in each population. For example, five QTL for leaf senescence were mapped in the *Ct-1*×*Col-0* population, three in the *Cvi-0*×*Col-0* population and two in the *Bur-0*×*Col-0* population (Fig. 1D–F). Overall, the composite interval mapping method uncovered 101 QTL for the ten studied traits in the three RIL populations. The data for each QTL are shown in Supplementary Table S1 (available at *JXB* online), including the original chromosomal position, confidence interval (CI), and proportion of phenotypic variance (*R* ^*2*^) explained. The number of QTL for each trait varied from zero to seven per population, and each QTL explained 7–54% of the phenotypic variance. The use of common markers across the three different maps allowed us to directly compare the detected QTL in a single consensus map (Fig. 2).

A major QTL, named *Shoot Growth 1* (*SG1*), previously identified in the *Bur-0*×*Col-0* population by Vlad *et al.* (2010, in Supplementary Fig. S1B available at *JXB* online), was consistently detected on chromosome 5, for several yield-related traits in the *Bur-0*×*Col-0* and *Ct-1*×*Col-0* populations. The *SG1* locus accounted for the majority of the phenotypic variation for FT (*R* ^*2*^= 33%), HI (*R* ^*2*^=54%), Seeds (*R* ^*2*^=49%), Seed C% (*R* ^*2*^=35%), Seed N% (*R* ^*2*^=14%), and leaf senescence (*R* ^*2*^=11%) in the *Bur-0*×*Col-0* population, and for Seed C% (*R* ^*2*^=26%), Seed N% (*R* ^*2*^=18%), Seeds (*R* ^*2*^=17%), TGW% (*R* ^*2*^=9%), and RV% (*R* ^*2*^=11%) in the *Ct-1*×*Col-0* population (Supplementary Table S1 available at *JXB* online). The discovered effect of *SG1* on resource allocation in seeds has been validated using near isogenic lines differing only at the *SG1* locus (personal communication from O. Loudet, The Institut Jean-Pierre Bourgin, Versailles). The lines carrying the *Bur-0* allele showed significantly higher scores for the traits Rosette, Stem, Seeds, as well as a significantly higher Seed C% and lower Seed N%, compared with the lines carrying the *Col-0* allele, responsible for the defective shoot growth phenotype (Supplementary Table S2 available at *JXB* online). Owing to the strong effect of the *SG1* locus, its presence hampered the detection of other minor QTL, especially in the *Bur-0*×*Col-0* population.

### Meta-analysis to decipher hot spots

**Fig. 3.**
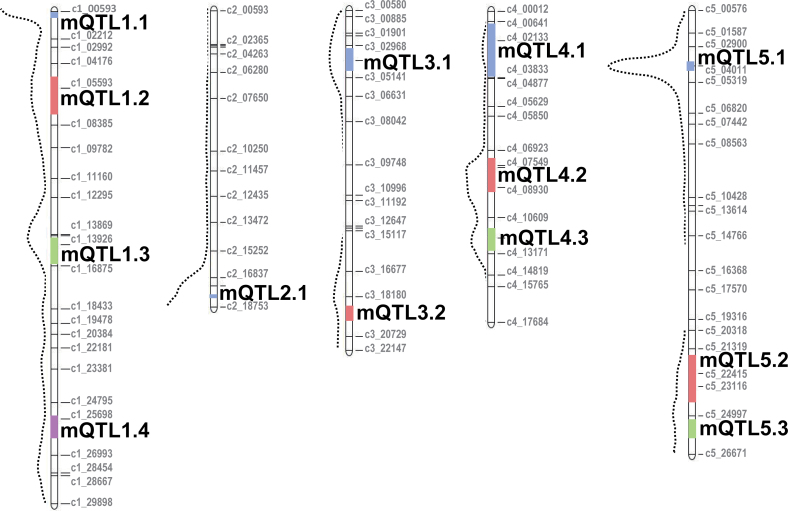
QTL Meta-analysis results in a synthetic genetic model with 13 metaQTL. MetaQTL are shown in coloured squares on the five chromosomes. Dash curves, named overview curves in Chardon *et al.* (2004), represent the density of detected QTL. (This figure is available in colour at *JXB* online.)

The 101 detected QTL were projected onto the same consensus map (Sosnowski *et al.*, 2012) and combined using a meta-analysis method. The meta-analysis resulted in a synthetic genetic model with 13 meta-QTL (Fig. 3, Table 4). These results are based on the hypothesis that the different traits are controlled by same genes, although we know that different QTL might overlap and be explained by different but close-by genes. Nevertheless, meta-analysis is a very good approach for identifying the most interesting loci (Table 4), in addition to the overview curve defined by Chardon *et al.* (2004), which represents the density of initial QTL detected (Fig. 3). The number of metaQTL identified on each chromosome varied from one on chromosome 2 to four on chromosome 1, with an average of 2.6 metaQTL per chromosome. The 13 metaQTL which form the synthetic genetic model are listed below and in Table 4:

**Table 4.** Meta-analysis results for ten traits related to leaf senescence and resource allocation in seeds. For each locus (metaQTL), populations and traits for which QTL were detected are indicated Estimated effects of each metaQTL on traits in each population are shown when the effect is significant. Blue and red highlight negative and positive effects, respectively, of the *Col-0* allele compared with the *Ct-1*, *Cvi-0*, and *Bur-0* alleles. The colour intensity corresponds to the strength of the effect: the stronger the effect, the darker the colour.

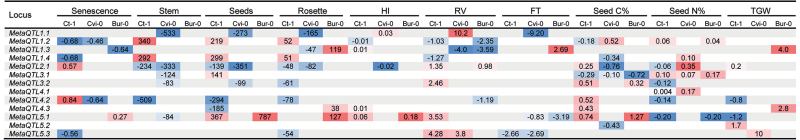

Traits are described in Table 1. HI, harvest index; RV, ratio between reproductive and vegetative organ weights; FT, flowering time; TGW, thousand grain weight

*MetaQTL1.1* is specific to the *Cvi-0*×*Col-0* population as all QTL clustered at this locus were only detected in this population (Table 4). The *Cvi-0* allele decreases plant biomass (–165mg, –533mg and –273mg for Rosette, Stem, and Seeds, respectively), induces early FT (–9.2 d) while it raises RV (+10.2). *MetaQTL1.1* explains in part the high correlation observed between these traits in the *Cvi-0*×*Col-0* population (Table 3). The effect of *metaQTL1.1* is consistent with the effect of a gene involved in FT.

*MetaQTL1.2* affects leaf senescence in *Ct-1*×*Col-0* and *Cvi-0*×*Col-0* populations. The *Ct-1* and *Cvi-0* alleles reduce leaf senescence compared with the *Col-0* allele (–0.68 and –0.46, respectively). At *metaQTL1.2*, the *Ct-1* allele increases plant biomass (+52mg, +340mg, and +219mg for Rosette, Stem, and Seeds, respectively), slightly reducing the HI (–0.01) and RV (–1.03), and resulting in lower Seed C% (–0.18%) and higher Seed N% (+0.06%). The *Cvi-0* allele has no effect on plant biomass but significantly increases Seed C% (+0.52%). These contrasting allelic effects on growth traits and resource allocation could be explained by either (i) two independent loci in *Ct-1*×*Col-0* and *Cvi-0*×*Col-0* populations with allele-specific effects on traits, (ii) two loci located under *metaQTL1.2* independently controlling leaf senescence and resource allocation, or (iii) a single locus with same allelic effect on leaf senescence but allele-specific effects on biomass and seed composition.

*MetaQTL1.3* was mainly detected in the *Bur-0*×*Col-0* population. The *Bur-0* allele decreases leaf senescence (–0.64), but increases Rosette (+119mg) and delays FT (+2.7 d). No effect on seed C% and N% was detected, but TGW is affected (+4.0mg). Contrasting effects on Rosette (–47mg) and the RV (–4.0) were measured in the *Cvi-0*×*Col-0* population.

*MetaQTL1.4* mainly affects leaf senescence and plant biomass in the *Ct-1*×*Col-0* population, whereas it is involved in seed composition traits in the *Cvi-0*×*Col-0* population. The *Ct-1* allele reduces leaf senescence (–0.68), increases plant biomass (+51mg, +299mg and +292mg for Rosette, Stem and Seeds respectively), slightly increasing the HI (+0.01) and decreasing the RV (–1.3), compared with the *Col-0* allele. The *Cvi-0* allele has no effect on plant growth but reduces Seed C% (–0.34%) and increases Seed N% (+0.10 %). The most likely explanation for these different effects is that *metaQTL1.4* clusters two independent loci.

*MetaQTL2.1* has contrasting effects on leaf senescence and plant biomass in the *Ct-1*×*Col-0* population. The *Ct-1* allele increases leaf senescence (+0.57), but reduces plant biomass (–48mg, –234mg and –139mg for Rosette, Stem, and Seeds, respectively), compared with the *Col-0* allele. *MetaQTL2.1* has similar effects on plant biomass in the *Cvi-0*×*Col-0* population, but no significant effect on leaf senescence was detected in this population. The *cvi-0* allele decreases plant biomass (–82mg, –333mg, and –351mg for Rosette, Stem, and Seeds, respectively), compared with the *Col-0* allele. Interestingly, *metaQTL2.1* has opposing effects on both Seed C% and Seed N% in the two populations. The *Ct-1* allele increases Seed C% (+0.25%), but reduces Seed N% (–0.06%) whereas the *Cvi-0* allele reduces Seed C% (–0.76%) but increases Seed N% (+0.35%), compared with the *Col-0* allele. Such opposite effects at *metaQTL2.1* could be explained by several hypotheses such as those described above for *metaQTL1.2*.

*MetaQTL3.1* is mainly involved in seed composition in the three populations. The *Ct-1*, *Cvi-0*, and *Bur-0* alleles reduce Seed C% (–0.29%, –0.10%, and –0.72%, respectively) and increase Seed N% (+0.10%, +0.07%, and +0.17%, respectively), compared with the *Col-0* allele. Allele-specific effects on Stem (–124mg) and Seeds (+141mg) were detected in the *Cvi-0*×*Col-0* and *Ct-1*×*Col-0* populations, respectively.

*MetaQTL3.2* is involved in the control of reproductive organs (Stem and Seeds) in the *Cvi-0*×*Col-0* population, but without a detected effect on seed composition. The *Cvi-0* allele reduces Stem (–83mg) and Seeds (–99mg) compared with the *Col-0* allele. In contrast, m*etaQTL3.2* affects only Rosette in the *Ct-1*×*Col-0* population, resulting in variation in the RV. This locus also dramatically affects Seed C% in the *Ct-1*×*Col-0* and *Bur-0*×*Col-0* populations. The *Ct-1* and *Bur-0* alleles increase Seed C% (respectively +0.51% and +0.32%), compared with the *Col-0* allele.

*MetaQTL4.1* has a weak effect on Seed N%, but no significant effect on Seed C% in the *Ct-1*×*Col-0* and *Cvi-0*×*Col-0* populations. The *Ct-1* and *Cvi-0* alleles increase Seed N% (+0.004% and +0.17%, respectively), compared with the *Col-0* allele.

*MetaQTL4.2* shows marked, but opposite effects on leaf senescence in the *Ct-1*×*Col-0* and *Cvi-0*×*Col-0* populations. The *Ct-1* allele increases leaf senescence (+0.84), whereas the *Cvi-0* allele reduces leaf senescence (–0.64), compared with the *Col-0* allele. Together with this effect on leaf senescence, the *Ct-1* allele decreases plant biomass (–78mg, –509mg, and –294mg for Rosette, Stem, and Seeds, respectively), shows higher Seed C% (+0.52%), and lower Seed N% (–0.14%), and reduced TGW (–0.8mg), compared with the *Col-0* allele.

*MetaQTL4.3* clusters QTL detected in the *Ct-1*×*Col-0* and *Bur-0*×*Col-0* populations. The *Ct-1* allele decreases Seeds (–185mg), slightly increases the HI (+0.01), and leads to seeds with high Seed C% (+0.43%), compared with the *Col-0* allele. The *Bur-0* allele increases Rosette (+38mg) and TGW (+2.8mg), compared with the *Col-0* allele. Such contrary effects suggest that *metaQTL4.3* clusters different loci from the two populations.

*MetaQTL5.1* is most likely the *SG1* locus, as previously mentioned. It has a dramatic effect on Seeds, HI, FT, Seed C%, and Seed N% in the *Ct-1*×*Col-0* and *Bur-0*×*Col-0* populations.

*MetaQTL5.2* is an incongruent locus clustering only two initial QTL. The *Ct-1* allele increases TGW (–1.7mg), whereas the *Cvi-0* allele reduces Seed C% (–0.43%), compared with the *Col-0* allele.

*MetaQTL5.3* clusters QTL detected in the *Ct-1*×*Col-0* and *Cvi-0*×*Col-0* populations. The *Ct-1* and *Cvi-0* alleles reduce FT (–2.66 d and –2.69 d, respectively) and increase the RV (+4.3 and +3.8, respectively), compared with the *Col-0* allele. The *Ct-1* allele also reduces leaf senescence (–0.56) and Rosette (–54mg), compared with the *Col-0* allele. The *metaQTL5.3* effect is consistent with the effect of a gene involved in FT.

### Candidate genes overlapping metaQTL

**Fig. 4.**
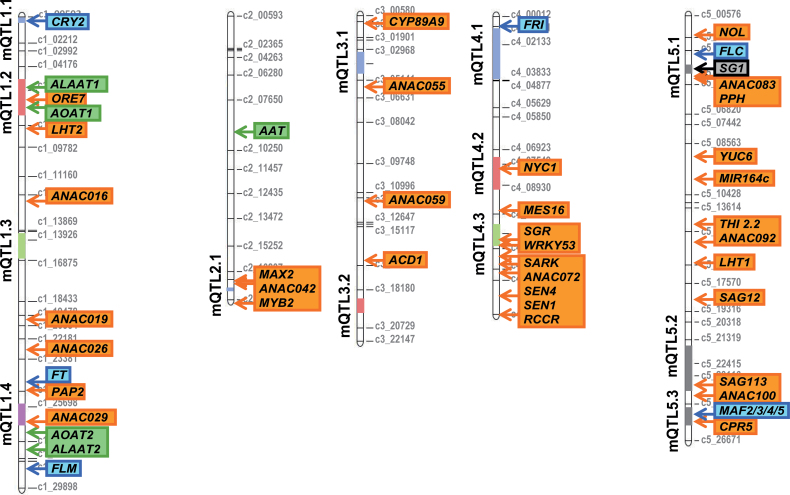
MetaQTL (mQTL) were compared with the position of candidate genes for flowering time, senescence, resource allocation and the *SG1* locus. (This figure is available in colour at *JXB* online.)

In addition to the co-localization between *SG1* and *metaQTL5.1*, several other overlaps were observed between detected metaQTL and genes known from the literature to be involved in flowering time, resource allocation, and leaf senescence, suggesting that they may be responsible for the phenotypic variation observed. Fig. 4 shows the metaQTL map with the localization of these candidate genes, which are also listed in Supplementary Table S3 (available at *JXB* online). M*etaQTL1.1* and *metaQTL5.3* overlapped with the *CRY2* (*CRYPTOCHROME 2*) and *MAF2/3/4/5* flowering time genes, respectively. Considering genes involved in leaf senescence and resource allocation processes, we also found several candidates which co-localized with metaQTL: *MetaQTL1.2* with the *ORE7* (*ORESARA 7*), *ALAAT1* (*ALANINE AMINOTRANSFERASE 1*), and *AOAT1* (*ALANINE-2-OXOGLUTARATE AMINOTRANSFERASE 1*) genes*; MetaQTL1.4* with the *ANAC029* gene; *MetaQTL4.2* with the *NYC1* (*NON-YELLOW COLORING 1*) gene; *MetaQTL4.3* with the *SGR* (*STAY-GREEN*) and *WRKY53* genes; and *MetaQTL5.2* with the *SAG113* (*SENESCENCE-ASSOCIATED GENE 113*) gene.

### Leaf senescence disrupts the negative correlation between Seed N% and Seeds

**Fig. 5.**
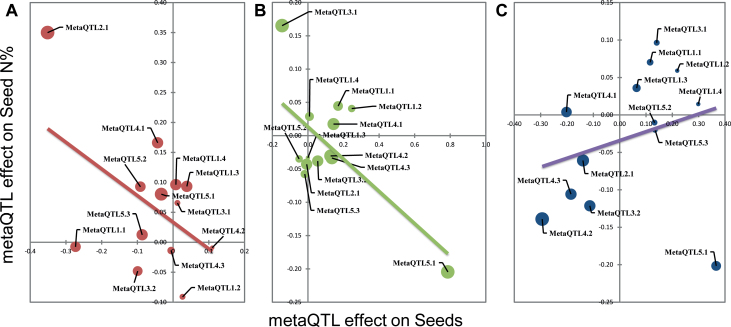
Effect of meta-QTL estimated on Seeds and Seed N% in the three RIL populations: *Cvi-0*×*Col-0* (A), *Bur-0*×*Col-0* (B), and *Ct-1*×*Col-0* (C). The size of the circle is proportional to the effect of meta-QTL on leaf senescence. (This figure is available in colour at *JXB* online.)

The seed N concentration is frequently negatively correlated to seed yield in crops (Uauy *et al.*, 2006, Gregersen *et al.*, 2013). A negative correlation was observed here in both the *Cvi-0*×*Col-0* and *Bur-0*×*Col-0* populations, but not in the *Ct-1*×*Col-0* population (Table 3). We studied the different relationships between Seed N% and Seeds on the basis of our genetic architecture model controlling these traits. Fig. 5 shows a scatter plot of all the estimated effects of the 13 metaQTL on Seeds and Seed N% (significant as well as non-significant effects). This figure shows that the estimated effects of the metaQTL on Seeds and on Seed N% are negatively correlated in the *Cvi-0*×*Col-0* (Fig. 5A) and *Bur-0*×*Col-0* (Fig. 5B) populations, with correlation coefficients of *r* ^*2*^=–0.50 and *r* ^*2*^=–0.67 respectively. This means that for most of the metaQTL, when they have a positive effect on one trait they have a negative effect on the other trait (Table 4). In contrast, the estimated effects of metaQTL on Seeds and Seed N% were positively correlated in the *Ct-1*×*Col-0* population (Fig. 5C). Although the correlation is low (*r* ^*2*^=+0.27) owing to the outlier *metaQTL5.1* (*SG1*) mentioned before, the alleles at the different metaQTL act both on Seeds and Seed N% and in the same direction (Table 4).

On the same scatter plot, the effects of the 13 metaQTL on leaf senescence are shown by the size of their plots (Fig. 5A–C). There was no relationship between leaf senescence and the two traits Seeds and Seed N% in the *Cvi-0*×*Col-0* and *Bur-0*×*Col-0* populations (Table 3). However, in the *Ct-1*×*Col-0* population, the effect of the metaQTL on senescence is related to the positive correlation of metaQTL with Seeds and Seed N% (Fig. 5C). This suggests that the metaQTL with the weakest effects of the *Ct-1* allele on Seeds and Seed N% traits together have the strongest positive effects on leaf senescence. In contrast, the metaQTL with the strongest effects of the *Ct-1* allele on Seeds and Seed N% together have the most negative effects on leaf senescence. For example, the *Ct-1* allele at *metaQTL4.2* reduces Seeds (–294mg) and Seed N% (–0.14%), but increases leaf senescence (+0.84), whereas at *metaQTL1.2* it increases Seeds (+219mg) and Seed N% (+0.06%), but moderates leaf senescence (–0.68). This suggests that in the *Ct-1*×*Col-0* population the leaf senescence phenotype has reversed the negative correlation existing between Seed N% and Seeds in the *Cvi-0*×*Col-0* and *Bur-0*×*Col-0* populations.

## Discussion

### Association between leaf senescence and resource allocation traits in *Arabidopsis*

During plant development, leaf senescence occurring during vegetative growth has an impact on rosette size; plants showing strong senescence phenotypes have small rosettes (Loudet *et al.*, 2003; Diaz *et al.*, 2006; Diaz *et al.*, 2008). However, the long-term effect of leaf senescence on resource allocation is largely unknown and to date none of the *Arabidopsis* studies on leaf senescence considered resource allocation to seeds. In the present study, leaf senescence was scored at the vegetative stage and compared with nine resource-allocation traits measured at seed maturity stage (Table 1) in three different RIL populations.

In the *Ct-1*×*Col-0* population, in which the differences between the values for the parental lines were the highest, leaf senescence is negatively correlated to final rosette weight, confirming our previous observation (Diaz *et al.*, 2006). Leaf senescence is also negatively correlated to reproductive organ growth and yield (Stem and Seeds; Table 3). Thus, leaf senescence seems to limit rosette development as well as the final plant development stages. However, because leaf senescence is positively correlated to the RV ratio (reproductive to vegetative biomass ratio; Table 3), its negative impact on growth seems to be stronger on rosettes than reproductive organs. Furthermore, leaf senescence also correlates positively with Seed C% and negatively with Seed N%, which is similar to the effects on seed resource allocation found in cereals (Table 3; Uauy *et al.*, 2006). No correlation between seed composition or leaf senescence and flowering time was found in the *Ct-1*×*Col-0* population showing that N and C seed allocation is most likely only related to leaf senescence and to the size of the source organs (rosette and stems) rather than to the duration of grain filling. Therefore, our results suggest that in the *Ct-1*×*Col-0* population sequential senescence decreases the nitrogen remobilization efficiency from the rosette to the reproductive organs and alters Seed N%.

In contrast, in the *Cvi-0*×*Col-0* and *Bur-0*×*Col-0* populations, leaf senescence is not correlated to seed yield (Seeds) or to seed composition (Seed C% and Seed N% in Table 3). Leaf senescence is, however, negatively correlated to seed weight (TGW) in both populations, suggesting that the seeds of early senescing genotypes are smaller than those of late senescing genotypes, but that they produce a greater number of seeds so that overall seed yield remains unchanged. The negative correlation between rosette biomass and leaf senescence found in the *Bay-0*×*Shahdara* (Diaz *et al.* 2006) and *Ct-1*×*Col-0* (present work) populations, is also found in the *Bur-0*×*Col-0* population, which also shows a negative correlation between leaf senescence and flowering time, and a positive correlation between leaf senescence and RV.

Finally, as previously described for various accessions (Masclaux-Daubresse and Chardon, 2011), no correlation between the HI and leaf senescence was detected for any of the three RIL populations (Table 3). The positive relationship between HI, Seed C%, and yield (Seeds) with the negative correlation between HI and flowering time suggest that seed production and seed carbon filling are mainly due to the length of the reproductive stage.

These variations in the relationship between leaf senescence, nitrogen use efficiency (Diaz *et al.*, 2006), rosette biomass, yield, and flowering related-traits suggest distinct and complex genetic control of leaf senescence in the different populations. This may explain why it was difficult to find clear links between all these traits when we analysed several accessions in our previous study (Masclaux-Daubresse and Chardon, 2011).

### The genetic basis of leaf senescence and resource allocation in seeds

To decipher the genetic architecture of the studied traits, we performed QTL mapping in the three populations, resulting in the detection of 101 QTL. Using a meta-analysis method, we built a synthetic genetic model with 13 metaQTL clustering the initial QTL. Correlations between phenotypic traits could then be genetically analysed in light of metaQTL with pleotropic effects. Phenotypic co-variation between the different plant organs (Rosette, Stem, and Seeds) is supported by metaQTL clustering QTL for the three traits: *metaQTL1.2*, *metaQT1.4*, *metaQT2.1*, and *metaQTL4.2* in the *Ct-1*×*Col-0* population, and *metaQTL1.1* and *metaQTL2.1* in the *Cvi-0*×*Col-0* population. Indeed, in these two populations, overall plant biomass is controlled by different loci revealing the complexity of the genetic architecture for these traits in *Arabidopsis*. Co-localization of QTL involved in growth of vegetative and reproductive organs was previously reported in maize and *Brassica olacera* (Lan and Paterson, 2001; Dignat *et al.*, 2013). In the *Bur-0*×*Col-0* population, no QTL for Stem were detected, explaining the absence of such a global metaQTL effect.

### MetaQTL for HI, RV, and FT

The timely transition from vegetative to reproductive growth is critical for successful resource allocation in seeds. In this study, three traits were investigated to monitor seed resource allocation: the HI, the ratio between reproductive organ weight and vegetative organ weight (RV) and the FT (Table 1). In our genetic model, in addition to the *SG1* locus, two major loci for FT, *metaQTL1.1* and *metaQTL5.3*, were identified on chromosomes 1 and 5, respectively (Fig. 4 and Table 4). At these two loci, metaQTL also cluster QTL for HI and/or RV, both with an opposite allelic effect compared with FT. Three other loci, *metaQTL1.2*, *metaQTL2.1* and *metaQTL4.2*, show significant effects mainly on the RV and to a lesser extent on the HI, but no significant effect on FT (Table 4). Therefore it seems that the negative correlation found between the HI and FT and discussed in the previous paragraph is mainly due to *metaQTL1.1* and *metaQTL5.3.* The three other loci, *metaQTL1.2*, *metaQTL2.1*, and *metaQTL4.2*, suggest that the development of reproductive organs (stem and seeds) can be independent of flowering time. Interestingly, similar results have been reported in a QTL study for stem branching in *Arabidopsis* in which the authors found that only three of the eight mapped stem branching QTL co-localized with flowering time genes (Huang *et al.*, 2013).

### MetaQTL for leaf senescence

Seven metaQTL clustered the initial QTL for leaf senescence. Only three also grouped initial QTL for FT in the same population: *metaQTL5.1-SG1*, *metaQTL1.3*, and *metaQTL5.3* (Table 4). Furthermore, no significant QTL for seed composition (Seed C% and Seed N%) was associated with these last two loci. This relationship is consistent with regulation of flowering time by leaf senescence previously reported in the *Bay*×*Shadhara* population (Wingler *et al.*, 2010). The four other metaQTL, which cluster QTL for leaf senescence and exclude QTL for flowering time (*metaQTL1.2*, *metaQTL1.4*, *metaQTL2.1*, and *metaQTL4.2*) encompass QTL for plant biomass (Rosette, Stem, and Seeds) and seed composition (Seed C% and Seed N%, Table 4). For all these loci, their effects on leaf senescence are negatively correlated to plant biomass and Seed N%, but positively correlated to Seed C%. These loci support the phenotypic correlation between these traits observed in the *Ct-1*×*Col-0* population. In this *Ct-1*×*Col-0* population, we showed that the relationship between QTL effects on seed yield (Seeds) and Seed N% is the inverse of that commonly observed in crops (Oury and Godin, 2007; Ciampitti and Vyn, 2013) and to that found in the other two populations (Fig. 5). The absence of a negative correlation between seed N% and yield in the *Ct-1*×*Col-0* population is probably due to interference of the leaf senescence effect on nitrogen-remobilization efficiency. This result shows that it is possible to find senescence-related genes in *Arabidopsis* that can disrupt the antagonistic effects of leaf senescence on grain yield and N content in seeds usually described in crops (Gregersen *et al.*, 2013).

### MetaQTL for seed composition

Among the three metaQTL (*metaQTL3.1*, *metaQTL3.2*, and *metaQTL4.1*) clustering QTL for seed composition (Seed C% and/or Seed N%), but not for flowering time or leaf senescence (Table 4), we found that two (*metaQTL3.1* and *metaQTL4.1*) co-localize with QTL for seed oil content previously reported in other *Arabidopsis* populations by Hobbs *et al.* (2004) and O’Neill *et al.* (2012). This suggests that these two genomic regions are indeed involved in seed storage regulation. At the moment, no obvious candidate genes can be assigned to these loci. Nevertheless, the genes uncovered by these metaQTL should be good targets for plant breeding as these loci have low pleiotropic morphological effects. *MetaQTL4.1* is of particular interest because it only affects Seed N% without affecting Seed C% in two populations (*Ct-1*×*Col-0*, *Cvi-0*×*Col-0*), even if the negative correlation between Seed C% and Seed N% was still observed (Table 3). The fine mapping of this locus will help understand its role in seed composition.

### Diverse candidate genes underlie the genetic model

The relationship between traits at metaQTL may correspond to pleiotropic genes controlling leaf senescence and resource allocation, or to different closely linked genes. Considering genes involved in the regulation of flowering time, co-localization of *metaQTL1.1* and *metaQTL5.3* with *CRY2* and *MAF2/3/4/5*, respectively, is consistent with their effects. Indeed, *CRY2* and *MAF2/3/4/5* have been shown to play a role in regulating flowering time (Guo *et al.*, 1998) and similar co-localization results have been reported (Ungerer *et al.*, 2002; El-Lithy *et al.*, 2004; El-Lithy *et al.*, 2006; Simon *et al.*, 2008; Fournier-Level *et al.*, 2013). *CRY2* encodes the blue-light photoreceptor cryptochrome-2 shown to promote flowering in long-day photoperiods, which correspond to our experimental photoperiod (Guo *et al.*, 1998). *MetaQTL1.1* was only detected in the *Cvi-0*×*Col-0* population. The *Cvi-0 CRY2* allele is a natural variant that induces early flowering time (El-Assal *et al.*, 2001). Plants carrying the *Ct-1* and *Cvi-0* alleles at *metaQTL5.3* display an earlier FT with a concomitant enhanced RV compared with plants carrying the *Col-0* allele. The genome structure of *MAF2/3/4/5* genes, which were found to co-localize with *metaQTL5.3*, is complex and natural variation in this structure leads to accelerated flowering time (Caicedo *et al.*, 2009; Rosloski *et al.*, 2010). Further experiments are needed to determine if there is an effect on Seed C% in *maf2/3/4/5* mutants.

Five other regions where metaQTL co-localized with genes involved in leaf senescence processes and resource allocation are shown in Fig. 4. *MetaQTL1.2*, which affects both leaf senescence and plant biomass, overlaps with the *ORE7*, *ALAAT1*, and *AOAT1* genes*. ORE7* encodes a protein with an AT-hook DNA-binding motif. Such proteins are known to co-regulate gene transcription through modification of chromatin architecture and it has been shown that leaf longevity increases markedly in the *ore7* mutant (Lim *et al.*, 2007). *ALAAT1* encodes an alanine aminotransferase. In rice, the overexpression of a barley alanine aminotransferase (AlaAT) significantly increases plant biomass and grain yield by enhancing nitrogen-uptake efficiency (Shrawat *et al.*, 2008). The *AOAT1* gene codes for an alanine-2-oxoglutarate aminotransferase 1 and the rosette of the *aoat1* mutant is pale green and grows more slowly than wild type (Verslues *et al.*, 2007).

*MetaQTL1.4* overlaps with the *ANAC029* gene and shows a major effect on leaf senescence and plant biomass (Rosette, Stem, and Seeds) in the *Ct-1*×*Col-0* population. The *ANAC029* gene encodes a NAC family transcription factor, which is a key factor in the regulation of leaf senescence (Guo and Gan, 2006). As plants carrying the *Ct-1* allele at this loci showed significantly delayed senescence compared with plants carrying the *Col-*0 allele, as in *anac029* mutants, we hypothesize that the *Ct-1 ANAC029* allele is less efficient at regulating the senescence process. *In silico* comparison of ANAC029 proteins of the four accessions identified *Ct-1*-specific polymorphisms (http://signal.salk.edu/atg1001/3.0/gebrowser.php). However, more experiments are needed to confirm that these polymorphisms are indeed responsible for the effect *of MetaQTL1.4* on leaf senescence.

*MetaQTL4.2* overlaps with the *NYC1* gene, which encodes a chlorophyll *b* reductase involved in the degradation of chlorophyll *b* and LHCII (light harvesting complex II) and is annotated as a leaf-senescence-associated gene (Gepstein *et al.*, 2003). *NYC1* also plays a crucial role in seed maturation (Nakajima *et al.*, 2012). The described roles of *NYC1* are thus consistent with the effects of *metaQTL4.2* on leaf senescence and seed composition. *MetaQTL4.3* covers two senescence regulatory genes SGR and *WRKY53*. *SGR* is similar to the tomato senescence-inducible chloroplast stay-green protein 1 and is upregulated during the *Arabidopsis* life cycle, especially in senescing leaves (Ren *et al.*, 2007). The *WRKY53* transcription factor is also involved in the progression of leaf senescence (Koyama *et al.*, 2013). Surprisingly, *MetaQTL4.3* affects Seeds and Seed C% in the *Ct-1*×*Col-0* population but not leaf senescence, suggesting that other genes besides *SGR* and *WRKY53* are responsible for the phenotypic variation.

*MetaQTL5.2*, which affects Seed C% in *Cvi-0*×*Col-0* and TGW in the *Ct-1*×*Col-0* population, overlaps with the *SAG113* gene encoding a member of the PP2C (PROTEIN PHOSPHATASE 2C) family controlling dehydration in senescing leaves (Zhang and Gan, 2012). No effect of *SAG113* on seeds has been reported so far. However *SAG113* is known to be under the control of the AtNAP transcription factor, which is a homologue of the wheat *TtNAM-B1* gene known as a master regulator of grain protein content in wheat (Zhang and Gan, 2012).

In all cases, further experiments are required to determine whether these candidate genes control the phenotypic variations observed and to identify the molecular mechanisms involved (modification of the coding sequence, of the expression level or of epigenetic regulation).

## Supplementary Data

Supplementary data are available at *JXB* online.

Supplementary Table S1: List of QTL detected in the three RIL populations. QTL nomenclature refers to (i) population, using the name of the parent different from *Col-0*; (ii) the trait; (iii) the chromosome where it is localized; and (iv) if several QTL for one trait are on the same chromosome, the number, as such: Parent_trait_chromosome_number. For each QTL the chromosome, the position (corresponding to the LOD score peak) and its confidence interval (CI, left and right borders) are indicated. The additive effect indicates the mean effect on the trait of the replacement of two *Col-0* alleles by two *Ct-1, Cvi-0*, or *Bur-0* alleles at the QTL. *R* ^*2*^ represents the proportion of phenotypic variance of the trait explained by the QTL.

Supplementary Table S2: Validation of the effects of the *SG1* – *metaQTL5.2* on plant biomass and seed composition using near isogenic lines carrying either *Bur-0* or *Col-0* alleles. Means and SD are shown (*n*=12) and probability that trait values are different depending on *Col-0* or *Bur-0* alleles at *SG1* from T-tests.

Supplementary Table S3: List of candidate genes involved in senescence process, resource allocation and flowering time. Name gene, complete name gene and ATG code are recorded from TAIR (http://www.Arabidopsis.org/index.jsp). Gene positions are described by Chromosome, Position (the physical position on AGI map), and Marker code (estimated position onto the consensus genetic map by a homothetic projection).

Supplementary Data

## References

[CIT0001] Abu-Shakra SS Phillips DA Huffaker RC 1978 Nitrogen fixation and delayed leaf senescence in soybeans. Science 199, 973–975 17752368 10.1126/science.199.4332.973

[CIT0002] Arcade A Labourdette A Falque M Mangin B Chardon F Charcosset A Joets J 2004 BioMercator: integrating genetic maps and QTL towards discovery of candidate genes. Bioinformatics 20, 2324–2326 15059820 10.1093/bioinformatics/bth230

[CIT0003] Arends D Prins P Jansen RC Broman KW 2010 R/qtl: high-throughput multiple QTL mapping. Bioinformatics 26, 2990–2992 20966004 10.1093/bioinformatics/btq565 PMC2982156

[CIT0004] Ay N Irmler K Fischer A Uhlemann R Reuter G Humbeck K 2009 Epigenetic programming via histone methylation at *WRKY53* controls leaf senescence in *Arabidopsis thaliana* . The Plant Journal 58, 333–346 19143996 10.1111/j.1365-313X.2008.03782.x

[CIT0005] Balazadeh S Parlitz S Mueller-Roeber B Meyer RC 2008 Natural developmental variations in leaf and plant senescence in *Arabidopsis thaliana* . Plant Biology 10, 136–147 18721318 10.1111/j.1438-8677.2008.00108.x

[CIT0006] Balazadeh S Siddiqui H Allu AD Matallana-Ramirez LP Caldana C Mehrnia M Zanor MI Kohler B Mueller-Roeber B 2010 A gene regulatory network controlled by the NAC transcription factor ANAC092/AtNAC2/ORE1 during salt-promoted senescence. The Plant Journal 62, 250–264 20113437 10.1111/j.1365-313X.2010.04151.x

[CIT0007] Blanco A Mangini G Giancaspro A 2012 Relationships between grain protein content and grain yield components through quantitative trait locus analyses in a recombinant inbred line population derived from two elite durum wheat cultivars. Molecular Breeding 30, 79–92

[CIT0008] Borrell AK Hammer GL Douglas ACL 2000 Does maintaining green leaf area in *Sorghum* improve yield under drought? I. Leaf growth and senescence. Crop Science 40, 1026–1037

[CIT0009] Caicedo AL Richards C Ehrenreich IM Purugganan MD 2009 Complex rearrangements lead to novel chimeric gene fusion polymorphisms at the *Arabidopsis thaliana MAF2-5* flowering time gene cluster. Molecular Biology and Evolution 26, 699–711 19139056 10.1093/molbev/msn300

[CIT0010] Chardon F Virlon B Moreau L Falque M Joets J Decousset L Murigneux A Charcosset A 2004 Genetic architecture of flowering time in maize as inferred from quantitative trait loci meta-analysis and synteny conservation with the rice genome. Genetics 168, 2169–2185 15611184 10.1534/genetics.104.032375 PMC1448716

[CIT0011] Churchill GA Doerge RW 1994 Empirical threshold values for quantitative trait mapping. Genetics 138, 963–971 7851788 10.1093/genetics/138.3.963 PMC1206241

[CIT0012] Ciampitti IA Vyn TJ 2013 Grain nitrogen source changes over time in maize: A review. Crop Science 53, 366–377

[CIT0013] Diaz C Lemaitre T Christ A Azzopardi M Kato Y Sato F Morot-Gaudry J-F Le Dily F Masclaux-Daubresse C 2008 Nitrogen recycling and remobilization are differentially controlled by leaf senescence and development stage in *Arabidopsis* under low nitrogen nutrition. Plant Physiology 147, 1437–1449 18467460 10.1104/pp.108.119040 PMC2442554

[CIT0014] Diaz C Purdy S Christ A Morot-Gaudry JF Wingler A Masclaux-Daubresse C 2005 Characterization of markers to determine the extent and variability of leaf senescence in *Arabidopsis*. A metabolic profiling approach. Plant Physiology 138, 898–908 15923326 10.1104/pp.105.060764 PMC1150406

[CIT0015] Diaz C Saliba-Colombani V Loudet O Belluomo P Moreau L Daniel-Vedele F Morot-Gaudry J-F Masclaux-Daubresse C 2006 Leaf yellowing and anthocyanin accumulation are two genetically independent strategies in response to nitrogen limitation in *Arabidopsis thaliana* . Plant and Cell Physiology 47, 74–83 16284408 10.1093/pcp/pci225

[CIT0016] Dignat G Welcker C Sawkins M Ribaut JM Tardieu F 2013 The growths of leaves, shoots, roots and reproductive organs partly share their genetic control in maize plants. Plant, Cell & Environment 36, 1105–1119 10.1111/pce.12045 23190045

[CIT0017] El-Assal SE Alonso-Blanco C Peeters AJ Raz V Koornneef M 2001 A QTL for flowering time in *Arabidopsis* reveals a novel allele of *CRY2* . Nature Genetics 29, 435–440 11726930 10.1038/ng767

[CIT0018] El-Lithy ME Bentsink L Hanhart CJ 2006 New *Arabidopsis* recombinant inbred line populations genotyped using SNPWave and their use for mapping flowering-time quantitative trait loci. Genetics 172, 1867–1876 16361234 10.1534/genetics.105.050617 PMC1456291

[CIT0019] El-Lithy ME Clerkx EJM Ruys GJ Koornneef M Vreugdenhil D 2004 Quantitative trait locus analysis of growth-related traits in a new *Arabidopsis* recombinant inbred population. Plant Physiology 135, 444–458 15122039 10.1104/pp.103.036822 PMC429397

[CIT0020] Fischer AM 2012 The complex regulation of senescence. Critical Reviews in Plant Sciences 31, 124–147

[CIT0021] Fournier-Level A Wilczek AM Cooper MD 2013 Paths to selection on life history loci in different natural environments across the native range of *Arabidopsis thaliana* . Molecular ecology 22, 3552–3566 23506537 10.1111/mec.12285

[CIT0022] Gan S Amasino RM 1997 Making sense of senescence (molecular genetic regulation and manipulation of leaf senescence). Plant Physiology 113, 313–319 12223609 10.1104/pp.113.2.313 PMC158144

[CIT0023] Gepstein S Sabehi G Carp M-J Hajouj T Nesher MFO Yariv I Dor C Bassani M 2003 Large-scale identification of leaf senescence-associated genes. The Plant Journal 36, 629–642 14617064 10.1046/j.1365-313x.2003.01908.x

[CIT0024] Grbic B Bleecker AB 1996 An altered body plan is conferred on *Arabidopsis* plants carrying dominant alleles of two genes. Development 122, 2395–403 8756285 10.1242/dev.122.8.2395

[CIT0025] Gregersen PL Culetic A Boschian L Krupinska K 2013 Plant senescence and crop productivity. Plant Molecular Biology 82, 603–622 23354836 10.1007/s11103-013-0013-8

[CIT0026] Guo H Yang H Mockler TC Lin C 1998 Regulation of flowering time by *Arabidopsis* photoreceptors. Science 279, 1360–1363 9478898 10.1126/science.279.5355.1360

[CIT0027] Guo Y Gan S 2006 AtNAP, a NAC family transcription factor, has an important role in leaf senescence. The Plant Journal 46, 601–612 16640597 10.1111/j.1365-313X.2006.02723.x

[CIT0028] Hobbs DH Flintham JE Hills MJ 2004 Genetic control of storage oil synthesis in seeds of *Arabidopsis* . Plant Physiology 136, 3341–3349 15466222 10.1104/pp.104.049486 PMC523393

[CIT0029] Huang X Ding J Effgen S Turck F Koornneef M 2013 Multiple loci and genetic interactions involving flowering time genes regulate stem branching among natural variants of *Arabidopsis* . New Phytologist 199, 843–857 23668187 10.1111/nph.12306

[CIT0030] Hunkovà E Živčák M Olšovská K 2011 Leaf area duration of oilseed rape (*Brassica napus subsp. napus*) varieties and hybrids and its relationship to selected growth and productivity parameters. Journal of Central European Agriculture 12, 1–15

[CIT0031] Ismail AM Hall AE Ehlers JD 2000 Delayed-leaf-senescence and heat-tolerance traits mainly are independently expressed in cowpea. Crop Science 40, 1049–1055

[CIT0032] Kim JH Woo HR Kim J Lim PO Lee IC Choi SH Hwang D Nam HG 2009 Trifurcate feed-forward regulation of age-dependent cell death involving *miR164* in *Arabidopsis* . Science 323, 1053–1057 19229035 10.1126/science.1166386

[CIT0033] Koyama T Nii H Mitsuda N Ohta M Kitajima S Ohme-Takagi M Sato F 2013 A regulatory cascade involving class II *ETHYLENE RESPONSE FACTOR* transcriptional repressors operates in the progression of leaf senescence. Plant Physiology 162, 991–1005 23629833 10.1104/pp.113.218115 PMC3668086

[CIT0034] Lan TH Paterson AH 2001 Comparative mapping of QTLs determining the plant size of *Brassica oleracea* . Theoretical and Applied Genetics 103, 383–397

[CIT0035] Lim PO Kim Y Breeze E 2007 Overexpression of a chromatin architecture-controlling AT-hook protein extends leaf longevity and increases the post-harvest storage life of plants. The Plant Journal 52, 1140–1153 17971039 10.1111/j.1365-313X.2007.03317.x

[CIT0036] Loudet O Chaillou S Merigout P Talbotec J Daniel-Vedele F 2003 Quantitative trait loci analysis of nitrogen use efficiency in *Arabidopsis* . Plant Physiology 131, 345–358 12529542 10.1104/pp.102.010785 PMC166814

[CIT0037] Masclaux-Daubresse C Chardon F 2011 Exploring nitrogen remobilization for seed filling using natural variation in *Arabidopsis thaliana* . Journal of Experimental Botany 62, 2131–2142 21273332 10.1093/jxb/erq405 PMC3060690

[CIT0037a] McKhann IH Camilleri C Bérard A Bataillon T David LJ Reboud X Le Corre V Caloustian C Gut GI Brunel D. 2004 Nested core collections maximizing genetic diversity in Arabidopsis thaliana. The Plant Journal 38, 193–202 15053772 10.1111/j.1365-313X.2004.02034.x

[CIT0038] Miao Y Laun T Zimmermann P Zentgraf U 2004 Targets of the WRKY53 transcription factor and its role during leaf senescence in *Arabidopsis* . Plant Molecular Biology 55, 853–867 15604721 10.1007/s11103-004-2142-6

[CIT0039] Miao Y Smykowski A Zentgraf U 2008 A novel upstream regulator of WRKY53 transcription during leaf senescence in *Arabidopsis thaliana* . Plant Biology 10, 110–120 18721316 10.1111/j.1438-8677.2008.00083.x

[CIT0040] Nakajima S Ito H Tanaka R Tanaka A 2012 Chlorophyll *b* reductase plays an essential role in maturation and storability of *Arabidopsis* seeds. Plant Physiology 160, 261–273 22751379 10.1104/pp.112.196881 PMC3440204

[CIT0041] O’Neill CM Morgan C Hattori C 2012 Towards the genetic architecture of seed lipid biosynthesis and accumulation in *Arabidopsis thaliana* . Heredity 108, 115–123 21731053 10.1038/hdy.2011.54 PMC3262871

[CIT0042] Olsen AN Ernst HA Leggio LL Skriver K 2005 NAC transcription factors: structurally distinct, functionally diverse. Trends in Plant Science 10, 79–87 15708345 10.1016/j.tplants.2004.12.010

[CIT0043] Oury F-X Godin C 2007 Yield and grain protein concentration in bread wheat: how to use the negative relationship between the two characters to identify favourable genotypes? Euphytica 157, 45–57

[CIT0044] Ren G An K Liao Y Zhou X Cao Y Zhao H Ge X Kuai B 2007 Identification of a novel chloroplast protein AtNYE1 regulating chlorophyll degradation during leaf senescence in *Arabidopsis* . Plant Physiology 144, 1429–41 17468209 10.1104/pp.107.100172 PMC1914121

[CIT0045] Rosloski SM Jali SS Balasubramanian S Weigel D Grbic V 2010 Natural diversity in flowering responses of *Arabidopsis thaliana* caused by variation in a tandem gene array. Genetics 186, 263–76 20551443 10.1534/genetics.110.116392 PMC2940291

[CIT0046] Schippers JHM Jing H-C Hille J Dijkwel PP 2007 Developmental and hormonal control of leaf senescence. In: Gan S, ed. Senescence Processes in Plants. Oxford: Blackwell Publishing Ltd, 145–170

[CIT0047] Shrawat AK Carroll RT DePauw M Taylor GJ Good AG 2008 Genetic engineering of improved nitrogen use efficiency in rice by the tissue-specific expression of alanine aminotransferase. Plant Biotechnology Journal 6, 722–732 18510577 10.1111/j.1467-7652.2008.00351.x

[CIT0048] Simmonds NW 1995 The relation between yield and protein in cereal grain. Journal of the Science of Food and Agriculture 67, 309–315

[CIT0049] Simon M Loudet O Durand S Bérard A Brunel D Sennesal F-X Durand-Tardif M Pelletier G Camilleri C 2008 Quantitative trait loci mapping in five new large recombinant inbred line populations of *Arabidopsis thaliana* genotyped with consensus single-nucleotide polymorphism markers. Genetics 178, 2253–2264 18430947 10.1534/genetics.107.083899 PMC2323813

[CIT0050] Sosnowski O Charcosset A Joets J 2012 BioMercator V3: an upgrade of genetic map compilation and quantitative trait loci meta-analysis algorithms. Bioinformatics 28, 2082–2083 22661647 10.1093/bioinformatics/bts313 PMC3400960

[CIT0051] Thomas H Stoddart JL 1980 Leaf senescence. Annual Review of Plant Physiology 31, 83–111

[CIT0052] Tian L Chen ZJ 2001 Blocking histone deacetylation in *Arabidopsis* induces pleiotropic effects on plant gene regulation and development. Proceedings of the National Academy of Sciences, USA 98, 200–205 10.1073/pnas.011347998 PMC14568 11134508

[CIT0053] Uauy C Distelfeld A Fahima T Blechl A Dubcovsky J 2006 A NAC gene regulating senescence improves grain protein, zinc, and iron content in wheat. Science 314, 1298–1301 17124321 10.1126/science.1133649 PMC4737439

[CIT0054] Ungerer MC Halldorsdottir SS Modliszewski JL Mackay TFC Purugganan MD 2002 Quantitative trait loci for inflorescence development in *Arabidopsis thaliana* . Genetics 160, 1133–1151 11901129 10.1093/genetics/160.3.1133 PMC1462026

[CIT0055] Verslues PE Kim Y-S Zhu J-K 2007 Altered ABA, proline and hydrogen peroxide in an *Arabidopsis* glutamate:glyoxylate aminotransferase mutant. Plant Molecular Biology 64, 205–217 17318317 10.1007/s11103-007-9145-z

[CIT0056] Vlad D Rappaport F Simon M Loudet O 2010 Gene transposition causing natural variation for growth in *Arabidopsis thaliana* . PLoS Genetics 6, e1000945 20485571 10.1371/journal.pgen.1000945 PMC2869320

[CIT0057] Wingler A Purdy SJ Edwards S-A Chardon F Masclaux-Daubresse C 2010 QTL analysis for sugar-regulated leaf senescence supports flowering-dependent and -independent senescence pathways. New Phytologist 185, 420–433 19878465 10.1111/j.1469-8137.2009.03072.x

[CIT0058] Zavaleta-Mancera HA Thomas BJ Thomas H Scott IM 1999 Regreening of senescent *Nicotiana* leaves: II. Redifferentiation of plastids. Journal of Experimental Botany 50, 1683–1689

[CIT0059] Zentgraf U Laun T Miao Y 2010 The complex regulation of *WRKY53* during leaf senescence of *Arabidopsis thaliana* . European Journal of Cell Biology 89, 133–137 20004496 10.1016/j.ejcb.2009.10.014

[CIT0060] Zhang K Gan S-S 2012 An abscisicacid-AtNAP transcription factor-SAG113 protein phosphatase 2C regulatory chain for controlling dehydration in senescing *Arabidopsis* leaves. Plant Physiology 158, 961–969 22184656 10.1104/pp.111.190876 PMC3271781

