# Supplementary material for: QTL meta-analysis in Arabidopsis reveals an interaction between leaf senescence and resource allocation to seeds
Source: J Exp Bot. 2014 Apr 1;65(14):3949–62. doi: 10.1093/jxb/eru125 (PMC4106442; doi:10.1093/jxb/eru125)
Supplement: Supplementary Data [file supp_65_14_3949__index.html]

QTL meta-analysis in Arabidopsis reveals an interaction between leaf senescence and resource allocation to seeds — QTL meta-analysis in Arabidopsis reveals an interaction between leaf senescence and resource allocation to seeds — QTL meta-analysis in Arabidopsis reveals an interaction between leaf senescence and resource allocation to seeds — Supplementary Data 

# QTL meta-analysis in *Arabidopsis* reveals an interaction between leaf senescence and resource allocation to seeds

## Supplementary Data

Data files

**Files in this Data Supplement:**

- Supplementary Data - Supplementary Data
