# Supplementary material for: QTL meta-analysis in Arabidopsis reveals an interaction between leaf senescence and resource allocation to seeds
Source: J Exp Bot. 2014 Apr 1;65(14):3949–62. doi: 10.1093/jxb/eru125 (PMC4106442; doi:10.1093/jxb/eru125)
Supplement: Supplementary Data [file supp_eru125_jexbot116327_file001.pdf]

Supplemental TableS1: List of QTL detected in the three RIL populations. QTL name refers to the population using name of the parent different from Col-0, the trait, the chromosome where it is localized and if several QTL for one trait on the same chromosome, the number as such: Parent_trait_chromosome_number. For each QTL the chromosome, the position (corresponding to the LOD score peak) and its confidence interval (CI, left and right borders) are indicated. The additive effect represents the mean effect on the trait of the replacement of two Col-0 alleles by two Ct-1, Cvi-0 or Bur-0 alleles at the QTL. R^2 represents the proportion of phenotypic variance of the trait explained by the QTL.

QTL	Trait	Population	Chromosome	Positon (cM)	CI (cM) left border	CI (cM) right border2	R^2 (%)
Cvi_FT_1	FT	Cvi-0xCol-0	1	0.0	0.0	6.0	43
Cvi_RV_1.1	RV	Cvi-0xCol-0	1	0.0	0.0	6.0	39
Cvi_Rosette_1.1	Rosette	Cvi-0xCol-0	1	0.0	0.0	6.0	15
Cvi_Seeds_1	Seeds	Cvi-0xCol-0	1	0.0	0.0	6.0	28
Cvi_Stem_1	Stem	Cvi-0xCol-0	1	0.0	0.0	6.0	48
Ct_HI_1.1	HI	Ct-1xCol-0	1	10.2	0.0	31.6	8
Ct_Seed%N_1	Seed N%	Ct-1xCol-0	1	10.2	0.0	36.7	12
Cvi_HI_1	HI	Cvi-0xCol-0	1	10.9	0.0	17.9	19
Bur_Seed%N_1	Seed N%	Bur-0xCol-0	1	17.8	8.3	34.6	9
Ct_Rosette_1.1	Rosette	Ct-1xCol-0	1	21.8	0.0	31.6	16
Ct_Seeds_1.1	Seeds	Ct-1xCol-0	1	21.8	0.0	82.9	8
Ct_Stem_1.1	Stem	Ct-1xCol-0	1	21.8	0.0	31.6	13
Cvi_Senes_1	Leaf senescence	Cvi-0xCol-0	1	24.6	17.9	37.5	15
Cvi_Seed%C_1.1	Seed C%	Cvi-0xCol-0	1	24.6	17.9	37.5	14
Cvi_Seed%N_1	Seed N%	Cvi-0xCol-0	1	24.6	6.0	37.5	24
Ct_Senes_1.1	Leaf senescence	Ct-1xCol-0	1	31.6	15.4	36.7	10
Ct_Seed%C_1	Seed C%	Ct-1xCol-0	1	31.6	10.2	36.7	11
Bur_FT_1	FT	Bur-0xCol-0	1	42.0	34.6	50.8	32
Bur_Senes_1	Leaf senescence	Bur-0xCol-0	1	42.0	34.6	50.8	28
Bur_RV_1	RV	Bur-0xCol-0	1	42.0	34.6	50.8	27
Bur_Rosette_1	Rosette	Bur-0xCol-0	1	42.0	34.6	50.8	16
Bur_TGW_1	TGW	Bur-0xCol-0	1	42.0	34.6	50.8	38
Cvi_Rosette_1.2	Rosette	Cvi-0xCol-0	1	49.1	24.6	82.2	7
Cvi_RV_1.2	RV	Cvi-0xCol-0	1	64.7	54.4	71.9	12
Ct_HI_1.2	HI	Ct-1xCol-0	1	69.2	61.3	73.9	14
Ct_RV_1	RV	Ct-1xCol-0	1	82.9	73.9	102.2	12
Ct_Rosette_1.2	Rosette	Ct-1xCol-0	1	82.9	73.9	89.3	14
Ct_Seeds_1.2	Seeds	Ct-1xCol-0	1	102.2	92.9	112.7	23
Ct_Stem_1.2	Stem	Ct-1xCol-0	1	102.2	92.9	112.7	7
Ct_Senes_1.2	Leaf senescence	Ct-1xCol-0	1	104.9	92.9	112.7	12
Cvi_Seed%C_1.2	Seed C%	Cvi-0xCol-0	1	130.6	71.9	138.1	9
Ct_RV_2	RV	Ct-1xCol-0	2	22.4	9.8	47.7	14
Bur_RV_2	RV	Bur-0xCol-0	2	28.5	9.3	55.8	7
Ct_TGW_2	TGW	Ct-1xCol-0	2	47.7	22.4	60.9	7
Ct_Seed%C_2	Seed C%	Ct-1xCol-0	2	66.9	47.7	70.8	17
Ct_Seeds_2	Seeds	Ct-1xCol-0	2	66.9	60.9	70.8	11
Ct_Senes_2	Leaf senescence	Ct-1xCol-0	2	70.8	37.9	70.8	10
Ct_Rosette_2	Rosette	Ct-1xCol-0	2	70.8	60.9	70.8	15
Ct_Seed%N_2	Seed N%	Ct-1xCol-0	2	70.8	47.7	70.8	10
Ct_Stem_2	Stem	Ct-1xCol-0	2	70.8	60.9	70.8	12
Cvi_Seeds_2	Seeds	Cvi-0xCol-0	2	80.1	77.4	86.9	34
Cvi_Stem_2	Stem	Cvi-0xCol-0	2	80.1	77.4	86.9	28
Cvi_HI_2	HI	Cvi-0xCol-0	2	86.9	77.4	86.9	9
Cvi_Rosette_2	Rosette	Cvi-0xCol-0	2	86.9	77.4	86.9	14
Cvi_Seed%C_2	Seed C%	Cvi-0xCol-0	2	86.9	77.4	86.9	31
Cvi_Seed%N_2	Seed N%	Cvi-0xCol-0	2	86.9	77.4	86.9	41
Bur_Seed%N_3	Seed N%	Bur-0xCol-0	3	5.7	0.0	14.5	19
Ct_Seed%C_3.1	Seed C%	Ct-1xCol-0	3	6.0	0.0	15.8	17
Ct_Seed%N_3.1	Seed N%	Ct-1xCol-0	3	6.0	0.0	15.8	19
Ct_Seeds_3	Seeds	Ct-1xCol-0	3	6.0	0.0	19.6	9
Bur_Seed%C_3.1	Seed C%	Bur-0xCol-0	3	8.0	0.0	11.1	20
Cvi_Seed%C_3	Seed C%	Cvi-0xCol-0	3	18.5	14.8	24.2	13
Cvi_Seed%N_3	Seed N%	Cvi-0xCol-0	3	18.9	0.0	30.4	14
Cvi_Stem_3.1	Stem	Cvi-0xCol-0	3	30.4	14.8	40.1	7
Bur_Seed%C_3.2	Seed C%	Bur-0xCol-0	3	54.2	42.0	65.5	20
Ct_Rosette_3	Rosette	Ct-1xCol-0	3	57.3	48.9	70.8	17
Ct_RV_3	RV	Ct-1xCol-0	3	63.6	25.9	70.9	9
Ct_Seed%C_3.2	Seed C%	Ct-1xCol-0	3	63.6	57.3	70.9	24
Ct_Seed%N_3.2	Seed N%	Ct-1xCol-0	3	63.6	48.9	74.2	14
Cvi_Seeds_3	Seeds	Cvi-0xCol-0	3	90.5	68.6	90.5	9
Cvi_Stem_3.2	Stem	Cvi-0xCol-0	3	90.5	75.8	90.5	11
Ct_Seed%N_4.1	Seed N%	Ct-1xCol-0	4	4.1	0.0	12.4	7
Cvi_Seed%N_4	Seed N%	Cvi-0xCol-0	4	19.1	6.5	28.6	11
Ct_Seed%C_4.1	Seed C%	Ct-1xCol-0	4	20.1	4.1	46.0	15
Bur_RV_4	RV	Bur-0xCol-0	4	25.6	0.0	40.9	7
Ct_Senes_4	Leaf senescence	Ct-1xCol-0	4	31.6	31.3	36.7	29
Bur_Rosette_4	Rosette	Bur-0xCol-0	4	35.7	0.0	40.9	8
Ct_Rosette_4	Rosette	Ct-1xCol-0	4	36.7	31.6	46.0	31
Ct_Seed%N_4.2	Seed N%	Ct-1xCol-0	4	36.7	31.6	46.0	31
Ct_Seeds_4	Seeds	Ct-1xCol-0	4	36.7	31.3	46.0	27

Ct_Stem_4	Stem	Ct-1xCol-0	4	36.7	31.6	46.0	27
Ct_TGW_4	TGW	Ct-1xCol-0	4	36.7	28.1	52.2	8
Bur_TGW_4	TGW	Bur-0xCol-0	4	38.7	30.9	40.9	27
Cvi_Senes_4	Leaf senescence	Cvi-0xCol-0	4	43.9	40.0	51.3	25
Ct_HI_4	HI	Ct-1xCol-0	4	52.2	31.3	62.6	7
Ct_Seed%C_4.2	Seed C%	Ct-1xCol-0	4	52.2	46.0	56.8	12
Ct_Seed%N_4.3	Seed N%	Ct-1xCol-0	4	56.8	52.2	62.6	8
Ct_FT_5.1	FT	Ct-1xCol-0	5	4.5	0.0	98.1	7
Bur_Rosette_5	Rosette	Bur-0xCol-0	5	4.6	0.0	9.1	19
Bur_FT_5	FT	Bur-0xCol-0	5	9.1	6.1	11.5	33
Bur_HI_5	HI	Bur-0xCol-0	5	9.1	6.1	11.5	54
Bur_Seed%C_5	Seed C%	Bur-0xCol-0	5	9.1	6.1	11.5	35
Bur_Seed%N_5	Seed N%	Bur-0xCol-0	5	9.1	4.6	11.5	14
Bur_Seeds_5	Seeds	Bur-0xCol-0	5	9.1	6.1	11.5	49
Bur_Senes_5	Leaf senescence	Bur-0xCol-0	5	11.5	6.1	20.4	11
Ct_HI_5	HI	Ct-1xCol-0	5	13.3	8.9	17.4	29
Ct_Seed%C_5	Seed C%	Ct-1xCol-0	5	13.3	8.9	17.4	26
Ct_Seed%N_5	Seed N%	Ct-1xCol-0	5	13.3	8.9	17.4	18
Ct_Seeds_5	Seeds	Ct-1xCol-0	5	13.3	8.9	17.4	17
Ct_TGW_5.1	TGW	Ct-1xCol-0	5	17.4	4.5	23.9	9
Ct_RV_5.1	RV	Ct-1xCol-0	5	23.9	17.4	32.1	11
Cvi_Stem_5	Stem	Cvi-0xCol-0	5	28.6	18.1	96.5	6
Ct_TGW_5.2	TGW	Ct-1xCol-0	5	82.5	75.4	98.1	8
Ct_Senes_5	Leaf senescence	Ct-1xCol-0	5	88.1	80.3	98.1	29
Ct_RV_5.2	RV	Ct-1xCol-0	5	88.1	82.5	98.1	37
Cvi_Seed%C_5	Seed C%	Cvi-0xCol-0	5	91.6	85.1	96.5	9
Ct_FT_5.2	FT	Ct-1xCol-0	5	98.1	88.1	98.1	22
Ct_Rosette_5	Rosette	Ct-1xCol-0	5	98.1	82.5	98.1	22
Cvi_TGW_5	TGW	Cvi-0xCol-0	5	106.5	91.6	112.8	7
Cvi_FT_5	FT	Cvi-0xCol-0	5	112.8	106.5	112.8	11
Cvi_RV_5	RV	Cvi-0xCol-0	5	112.8	106.5	112.8	14

Supplemental TableS2: Validation of the effects of the SG1 – metaQTL5.2 on plant biomass and seed composition using near isogenic lines carrying either Bur-0 or Col-0 alleles. Means and SD are shown (n=12) and probability that trait values are different depending on Col-0 or Bur-0 alleles at SG1 from T-tests.

	**rHIF (allele)**				
	***Col-0* (n=12)**		***Bur-0* (n=12)**		**T-test**
**Traits**	**mean**	**Sdt**	**mean**	**Sdt**	
Rosette	110	18	433	30	<0.0001
Stem	908	179	2376	175	<0.0001
Seeds	63	35	1170	58	<0.0001
HI	8.2	2	8.284	1.1	*0.919*
RV	0.04	0.06	0.30	0.03	<0.0001
Seed %N	4.39	0.60	4.78	2.45	0.003
Seed %C	54.2	12.4	49.6	10.4	<0.0001

Supplemental TableS3: List of candidate genes involved in senescence process, resource allocation and flowering time. Name gene, complete name gene and ATG code are recorded from TAIR (http://www.arabidopsis.org/index.jsp). Gene positions are described by Chromosome, Position (the physical position on AGI map), and Marker code (estimated position onto the consensus genetic map by a homothetic projection).

Process	Name Gene	Complete Name gene	ATG code	Chromosome	Position	Marker code	Reference
Ressource allocation	ALAAT1	ALANINE AMINOTRANSFERASE 1	AT1G17290	c1	5922630	c1_05922	Shrawat AK, Carroll RT, DePauw M, Taylor GJ and Good AG. 2008. Genetic engineering of improved nitrogen use efficiency in rice by the tissue-specific expression of alanine aminotransferase. Plant biotechnology journal 6, 722-32.
Ressource allocation	AOAT1	ALANINE-2-OXOGLUTARATE AMINOTRANSFERASE 1	AT1G23310	c1	8268416	c1_08268	Verslues PE, Kim Y-S and Zhu J-K. 2007. Altered ABA, proline and hydrogen peroxide in an Arabidopsis glutamate:glyoxylate aminotransferase mutant. Plant Molecular Biology 64, 205-17.
Ressource allocation	AOAT2	ALANINE-2-OXOGLUTARATE AMINOTRANSFERASE 2	AT1G70580	c1	26612719	c1_26612	Liepman AH and Olsen LJ. 2003. Alanine Aminotransferase Homologs Catalyze the Glutamate:Glyoxylate Aminotransferase Reaction in Peroxisomes of Arabidopsis. Plant Physiology 131, 215-27.
Ressource allocation	ALAAT2	ALANINE AMINOTRANSFERASE 2	AT1G72330	c1	27233475	c1_27233	Shrawat AK, Carroll RT, DePauw M, Taylor GJ and Good AG. 2008. Genetic engineering of improved nitrogen use efficiency in rice by the tissue-specific expression of alanine aminotransferase. Plant biotechnology journal 6, 722-32.
Ressource allocation	AAT	ASPARTATE AMINOTRANSFERASE	AT2G22250	c2	9457810	c2_09457	de la Torre F, Santis LD, Suárez MF, Crespillo R and Cánovas FM. 2006. Identification and functional analysis of a prokaryotic-type aspartate aminotransferase: implications for plant amino acid metabolism. The Plant Journal 46, 414-25.
Leaf senesence	ORE7	ORESARA 7	AT1G20900	c1	7272556	c1_07272	Lim PO, Kim Y, Breeze E et al. 2007. Overexpression of a chromatin architecture-controlling AT-hook protein extends leaf longevity and increases the post-harvest storage life of plants. The Plant Journal 52, 1140-53.
Leaf senesence	LHT2	LYSINE HISTIDINE TRANSPORTER 2	AT1G24400	c1	8651407	c1_08651	Hirner A, Ladwig F, Stransky H, Okumoto S, Keinath M, Harms A, Frommer WB and Koch W. 2006. Arabidopsis LHT1 Is a High-Affinity Transporter for Cellular Amino Acid Uptake in Both Root Epidermis and Leaf Mesophyll. The Plant Cell Online 18, 1931-46.
Leaf senesence	ANAC016	NAC DOMAIN CONTAINING PROTEIN 16	AT1G34180	c1	12448726	c1_12448	Kim Y-S, Sakuraba Y, Han S-H, Yoo S-C and Paek N-C. 2013. Mutation of the Arabidopsis NAC016 Transcription Factor Delays Leaf Senescence. Plant and Cell Physiology 54, 1660-72.
Leaf senesence	ANAC019	NAC DOMAIN CONTAINING PROTEIN 19	AT1G52890	c1	19697004	c1_19697	Hickman R, Hill C, Penfold CA et al. 2013. A local regulatory network around three NAC transcription factors in stress responses and senescence in Arabidopsis leaves. The Plant Journal 75, 26-39.
Leaf senesence	ANAC026	ARABIDOPSIS NAC DOMAIN CONTAINING PROTEIN 29	AT1G62700	c1	23216218	c1_23216	Yamaguchi M, Kubo M, Fukuda H and Demura T. 2008. VASCULAR-RELATED NAC-DOMAIN7 is involved in the differentiation of all types of xylem vessels in Arabidopsis roots and shoots. The Plant Journal 55, 652-64.
Leaf senesence	PAP2	PRODUCTION OF ANTHOCYANIN PIGMENT 2	AT1G66390	c1	24763957	c1_24763	Gao P, Xin Z and Zheng Z-L. 2008. The OSU1/QUA2/TSD2-Encoded Putative Methyltransferase Is a Critical Modulator of Carbon and Nitrogen Nutrient Balance Response in Arabidopsis. PLoS ONE 3, e1387.
Leaf senesence	ANAC029	ARABIDOPSIS NAC DOMAIN CONTAINING PROTEIN 29	AT1G69490	c1	26122139	c1_26122	Guo Y and Gan S. 2006. AtNAP, a NAC family transcription factor, has an important role in leaf senescence. The Plant Journal 46, 601-12.
Leaf senesence	MAX2	MORE AXILLARY BRANCHES 2	AT2G42620	c2	17756140	c2_17756	Shen H, Zhu L, Bu Q-Y and Huq E. 2012. MAX2 Affects Multiple Hormones to Promote Photomorphogenesis. Molecular Plant 5, 750-62.
Leaf senesence	NAC042	NAC DOMAIN CONTAINING PROTEIN 42	AT2G43000	c2	17880615	c2_17880	Wu A, Allu AD, Garapati P et al. 2012. JUNGBRUNNEN1, a Reactive Oxygen Species–Responsive NAC Transcription Factor, Regulates Longevity in Arabidopsis. The Plant Cell Online 24, 482-506.
Leaf senesence	MYB2	MYB DOMAIN PROTEIN 2	AT2G47190	c2	19376202	c2_19376	Guo Y and Gan S. 2011. AtMYB2 Regulates Whole Plant Senescence by Inhibiting Cytokinin-Mediated Branching at Late Stages of Development in Arabidopsis. Plant Physiology 156, 1612-19.
Leaf senesence	CYP89A9	CYTOCHROME P450, FAMILY 87, SUBFAMILY A, POLYPEPTIDE 9	AT3G03470	c3	824552	c3_00824	Christ B, Süssenbacher I, Moser S, Bichsel N, Egert A, Müller T, Kräutler B and Hörtensteiner S. 2013. Cytochrome P450 CYP89A9 Is Involved in the Formation of Major Chlorophyll Catabolites during Leaf Senescence in Arabidopsis. The Plant Cell Online 25, 1868-80.
Leaf senesence	NAC055	NAC DOMAIN CONTAINING PROTEIN 55	AT3G15500	c3	5234617	c3_05234	Hickman R, Hill C, Penfold CA et al. 2013. A local regulatory network around three NAC transcription factors in stress responses and senescence in Arabidopsis leaves. The Plant Journal 75, 26-39.
Leaf senesence	ANAC059	ARABIDOPSIS NAC DOMAIN CONTAINING PROTEIN 59	AT3G29035	c3	11033665	c3_11033	Kim Y-S, Sakuraba Y, Han S-H, Yoo S-C and Paek N-C. 2013. Mutation of the Arabidopsis NAC016 Transcription Factor Delays Leaf Senescence. Plant and Cell Physiology 54, 1660-72.
Leaf senesence	ACD1	ACCELERATED CELL DEATH 1	AT3G44880	c3	16383595	c3_16383	Liu F and Guo F-Q. 2013. Nitric Oxide Deficiency Accelerates Chlorophyll Breakdown and Stability Loss of Thylakoid Membranes during Dark-Induced Leaf Senescence in Arabidopsis. PLoS ONE 8, e56345.
Leaf senesence	NYC1	NON-YELLOW COLORING 1	AT4G13250	c4	7684234	c4_07684	Nakajima S, Ito H, Tanaka R and Tanaka A. 2012. Chlorophyll b reductase plays an essential role in maturation and storability of Arabidopsis seeds. Plant Physiology 160, 261-73.
Leaf senesence	MES16	METHYL ESTERASE 16	AT4G16690	c4	9392147	c4_09392	Christ B, Schelbert S, Aubry S, Süssenbacher I, Müller T, Kräutler B and Hörtensteiner S. 2012. MES16, a Member of the Methylesterase Protein Family, Specifically Demethylates Fluorescent Chlorophyll Catabolites during Chlorophyll Breakdown in Arabidopsis. Plant Physiology 158, 628-41.
Leaf senesence	SGR	STAY-GREEN	AT4G22920	c4	12016525	c4_12016	Ren G, An K, Liao Y, Zhou X, Cao Y, Zhao H, Ge X and Kuai B. 2007. Identification of a Novel Chloroplast Protein AtNYE1 Regulating Chlorophyll Degradation during Leaf Senescence in Arabidopsis. Plant Physiology 144, 1429-41.
Leaf senesence	WRKY53		AT4G23810	c4	12392370	c4_12392	Koyama T, Nii H, Mitsuda N, Ohta M, Kitajima S, Ohme-Takagi M and Sato F. 2013. A regulatory cascade involving class II ETHYLENE RESPONSE FACTOR transcriptional repressors operates in the progression of leaf senescence. Plant Physiology 162, 991-1005.
Leaf senesence	ANAC072	ARABIDOPSIS NAC DOMAIN CONTAINING PROTEIN 72	AT4G27410	c4	13707900	c4_13707	Hickman R, Hill C, Penfold CA et al. 2013. A local regulatory network around three NAC transcription factors in stress responses and senescence in Arabidopsis leaves. The Plant Journal 75, 26-39.
Leaf senesence	SEN4	SENESCENCE 4	AT4G30270	c4	14819192	c4_14819	Oh SA, Lee SY, Chung I, Lee C-H and Nam HG. 1996. A senescence-associated gene of Arabidopsis thaliana is distinctively regulated during natural and artificially induced leaf senescence. Plant Molecular Biology 30, 739-54.

Leaf senesence	SARK	SENESCENCE-ASSOCIATED RECEPTOR-LIKE KINASE	AT4G30520	c4	14908000	c4_14908	Xu F, Meng T, Li P, Yu Y, Cui Y, Wang Y, Gong Q and Wang NN. 2011. A Soybean Dual-Specificity Kinase, GmSARK, and Its Arabidopsis Homolog, AtSARK, Regulate Leaf Senescence through Synergistic Actions of Auxin and Ethylene. Plant Physiology 157, 2131-53.
Leaf senesence	SEN1	ARABIDOPSIS THALIANA SENESCENCE 1	AT4G35770	c4	16944941	c4_16944	Fischer-Kilbienski I, Miao Y, Roitsch T, Zschiesche W, Humbeck K and Krupinska K. 2010. Nuclear targeted AtS40 modulates senescence associated gene expression in Arabidopsis thaliana during natural development and in darkness. Plant Molecular Biology 73, 379-90.
Leaf senesence	RCCR	RED CHLOROPHYLL CATABOLITE REDUCTASE	AT4G37000	c4	17442612	c4_17442	Sakuraba Y, Schelbert S, Park S-Y, Han S-H, Lee B-D, Andrès CB, Kessler F, Hörtensteiner S and Paek N-C. 2012. STAY-GREEN and Chlorophyll Catabolic Enzymes Interact at Light-Harvesting Complex II for Chlorophyll Detoxification during Leaf Senescence in Arabidopsis. The Plant Cell Online 24, 507-18.
Leaf senesence	NOL	NYC1-LIKE	AT5G04900	c5	1434774	c5_01434	Nakajima S, Ito H, Tanaka R and Tanaka A. 2012. Chlorophyll b reductase plays an essential role in maturation and storability of Arabidopsis seeds. Plant Physiology 160, 261-73.
Leaf senesence	ANAC083	NAC DOMAIN CONTAINING PROTEIN 83	AT5G13180	c5	4196572	c5_04196	Seo PJ and Park C-M. 2011. Signaling linkage between environmental stress resistance and leaf senescence in Arabidopsis. Plant signaling & behavior 6, 1564-66.
Leaf senesence	PPH	PHEOPHYTINASE	AT5G13800	c5	4451837	c5_04451	Schelbert S, Aubry S, Burla B, Agne B, Kessler F, Krupinska K and Hörtensteiner S. 2009. Pheophytin Pheophorbide Hydrolase (Pheophytinase) Is Involved in Chlorophyll Breakdown during Leaf Senescence in Arabidopsis. The Plant Cell Online 21, 767-85.
Leaf senesence	YUC6	YUCCA6	AT5G25620	c5	8935040	c5_08935	Kim JI, Murphy AS, Baek D, Lee S-W, Yun D-J, Bressan RA and Narasimhan ML. 2011. YUCCA6 over-expression demonstrates auxin function in delaying leaf senescence in Arabidopsis thaliana. Journal of experimental botany 62, 3981-92.
Leaf senesence	MIR164c	MICRORNA164C	AT5G27807	c5	9852664	c5_09852	Hasson A, Plessis A, Blein T, Adroher B, Grigg S, Tsiantis M, Boudaoud A, Damerval C and Laufs P. 2011. Evolution and Diverse Roles of the CUP-SHAPED COTYLEDON Genes in Arabidopsis Leaf Development. The Plant Cell Online 23, 54-68.
Leaf senesence	THI2.2	THIONIN 2.2	AT5G36910	c5	14561817	c5_14561	Epple P, Apel K and Bohlmann H. 1995. An Arabidopsis thaliana Thionin Gene Is Inducible via a Signal Transduction Pathway Different from That for Pathogenesis-Related Proteins. Plant Physiology 109, 813-20.
Leaf senesence	ANAC092	ARABIDOPSIS NAC DOMAIN CONTAINING PROTEIN 92	AT5G39610	c5	15858400	c5_15858	Balazadeh S, Kwasniewski M, Caldana C, Mehrnia M, Zanor MI, Xue G-P and Mueller-Roeber B. 2011. ORS1, an H2O2-Responsive NAC Transcription Factor, Controls Senescence in Arabidopsis thaliana. Molecular Plant 4, 346-60.
Leaf senesence	LHT1	LYSINE HISTIDINE TRANSPORTER 1	AT5G40780	c5	16323651	c5_16323	Liu G, Ji Y, Bhuiyan NH, Pilot G, Selvaraj G, Zou J and Wei Y. 2010. Amino Acid Homeostasis Modulates Salicylic Acid–Associated Redox Status and Defense Responses in Arabidopsis. The Plant Cell Online 22, 3845-63.
Leaf senesence	SAG12	SENESCENCE-ASSOCIATED GENE 12	AT5G45890	c5	18613259	c5_18613	Lohman KN, Gan S, John MC and Amasino RM. 1994. Molecular analysis of natural leaf senescence in Arabidopsis thaliana. Physiologia Plantarum 92, 322-28.
Leaf senesence	SAG113	SENESCENCE ASSOCIATED GENE 113	AT5G59220	c5	23894404	c5_23894	Zhang K, Xia X, Zhang Y and Gan S-S. 2012. An ABA-regulated and Golgi-localized protein phosphatase controls water loss during leaf senescence in Arabidopsis. The Plant Journal 69, 667-78.
Leaf senesence	ANAC100	NAC DOMAIN CONTAINING PROTEIN 100	AT5G61430	c5	24701122	c5_24701	Guo H-S, Xie Q, Fei J-F and Chua N-H. 2005. MicroRNA Directs mRNA Cleavage of the Transcription Factor NAC1 to Downregulate Auxin Signals for Arabidopsis Lateral Root Development. The Plant Cell Online 17, 1376-86.
Leaf senesence	CPR5	CONSTITUTIVE EXPRESSION OF PR GENES 5	AT5G64930	c5	25945712	c5_25945	Jing HC, Hebeler R, Oeljeklaus S et al. 2008. Early leaf senescence is associated with an altered cellular redox balance in Arabidopsis cpr5/old1 mutants. Plant Biology 10, 85-98.
Flowering time	CRY2	CRYPTOCHROME 2	AT1G04400	c1	1185550	c1_01185	Guo H, Yang H, Mockler TC and Lin C. 1998. Regulation of flowering time by Arabidopsis photoreceptors. Science 279, 1360-63.
Flowering time	GI	GIGANTEA	AT1G22770	c1	8061844	c1_08061	Levy YY and Dean C. 1998. The Transition to Flowering. The Plant Cell Online 10, 1973-89.
Flowering time	FT	FLOWERING LOCUS T	AT1G65480	c1	24331428	c1_24331	Kardailsky I, Shukla VK, Ahn JH, Dagenais N, Christensen SK, Nguyen JT, Chory J, Harrison MJ and Weigel D. 1999. Activation tagging of the floral inducer FT. Science 286, 1962-65.
Flowering time	FLM	FLOWERING LOCUS M	AT1G77080	c1	28955637	c1_28955	Levy YY and Dean C. 1998. The Transition to Flowering. The Plant Cell Online 10, 1973-89.
Flowering time	FRI	FRIGIDA	AT4G00650	c4	269026	c4_00269	Clarke JH and Dean C. 1994. Mapping FRI, a locus controlling flowering time and vernalization response in Arabidopsis thaliana. Mol. Gen. Genet. 242, 81-89.
Flowering time	TFL1	TERMINAL FLOWER 1	AT5G03840	c5	1024641	c5_01024	Shannon S and Meeks-Wagner DR. 1991. A mutation in the Arabidopsis TFL1 gene affects inflorescence meristem development. Plant Cell 3, 877-92.
Flowering time	FLC	FLOWERING LOCUS C	AT5G10140	c5	3173497	c5_03173	Bastow R, Mylne JS, Lister C, Lippman Z, Martienssen RA and Dean C. 2004. Vernalization requires epigenetic silencing of FLC by histone methylation. Nature 427, 164-67.
Flowering time	MAF2	MADS AFFECTING FLOWERING 2	AT5G65050	c5	25982254	c5_25982	Caicedo AL, Richards C, Ehrenreich IM and Purugganan MD. 2009. Complex rearrangements lead to novel chimeric gene fusion polymorphisms at the Arabidopsis thaliana MAF2-5 flowering time gene cluster. Molecular Biology and Evolution 26, 699-711.
Flowering time	MAF3	MADS AFFECTING FLOWERING 3	AT5G65060	c5	25987429	c5_25987	Caicedo AL, Richards C, Ehrenreich IM and Purugganan MD. 2009. Complex rearrangements lead to novel chimeric gene fusion polymorphisms at the Arabidopsis thaliana MAF2-5 flowering time gene cluster. Molecular Biology and Evolution 26, 699-711.
Flowering time	MAF4	MADS AFFECTING FLOWERING 4	AT5G65070	c5	25992243	c5_25992	Caicedo AL, Richards C, Ehrenreich IM and Purugganan MD. 2009. Complex rearrangements lead to novel chimeric gene fusion polymorphisms at the Arabidopsis thaliana MAF2-5 flowering time gene cluster. Molecular Biology and Evolution 26, 699-711.

**Flowering time**	MAF5	MADS AFFECTING FLOWERING 5	AT5G65080	c5	25997504	c5_25997	Caicedo AL, Richards C, Ehrenreich IM and Purugganan MD. 2009. Complex rearrangements lead to novel chimeric gene fusion polymorphisms at the Arabidopsis thaliana MAF2-5 flowering time gene cluster. Molecular Biology and Evolution 26, 699-711.
